# Photocatalytic Reduction of CO_2_ to CO in Aqueous Solution under Red-Light Irradiation by a Zn-Porphyrin-Sensitized Mn(I) Catalyst

**DOI:** 10.1021/acs.inorgchem.2c00091

**Published:** 2022-08-12

**Authors:** James Shipp, Simon Parker, Steven Spall, Samantha L. Peralta-Arriaga, Craig C. Robertson, Dimitri Chekulaev, Peter Portius, Simon Turega, Alastair Buckley, Rachael Rothman, Julia A. Weinstein

**Affiliations:** † Department of Chemistry, University of Sheffield, Sheffield S3 7HF, U.K.; ‡ Department of Chemistry, Sheffield Hallam University, Sheffield S1 1WB, U.K.; § Department of Physics and Astronomy, University of Sheffield, Sheffield S3 7RH, U.K.; ∥ Department of Chemical and Biological Engineering, University of Sheffield, Sheffield S1 3JD, U.K.

## Abstract

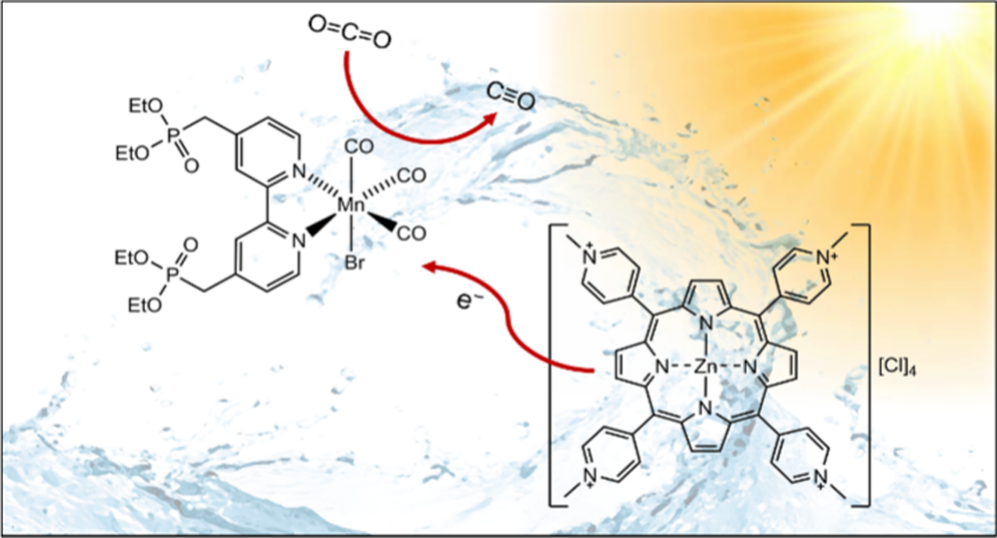

This work demonstrates photocatalytic CO_2_ reduction by a noble-metal-free photosensitizer-catalyst system in aqueous solution under red-light irradiation. A water-soluble Mn(I) tricarbonyl diimine complex, [MnBr(4,4′-{Et_2_O_3_PCH_2_}_2_-2,2′-bipyridyl)(CO)_3_] (**1**), has been fully characterized, including single-crystal X-ray crystallography, and shown to reduce CO_2_ to CO following photosensitization by tetra(*N*-methyl-4-pyridyl)porphyrin Zn(II) tetrachloride [Zn(TMPyP)]Cl_4_ (**2**) under 625 nm irradiation. This is the first example of **2** employed as a photosensitizer for CO_2_ reduction. The incorporation of −P(O)(OEt)_2_ groups, decoupled from the core of the catalyst by a −CH_2_– spacer, afforded water solubility without compromising the electronic properties of the catalyst. The photostability of the active Mn(I) catalyst over prolonged periods of irradiation with red light was confirmed by ^1^H and ^13^C{^1^H} NMR spectroscopy. This first report on Mn(I) species as a homogeneous photocatalyst, working in water and under red light, illustrates further future prospects of intrinsically photounstable Mn(I) complexes as solar-driven catalysts in an aqueous environment.

## Abstract 2

A Mn(I) bipyridyl tricarbonyl complex, where the diimine ligand is functionalized with water-solubilizing phosphonate ester groups, has been prepared and is shown to catalytically convert CO_2_ to CO in aqueous solution following photosensitization from a water-soluble Zn(II) porphyrin under red-light irradiation.

## Introduction

**Figure 1:**
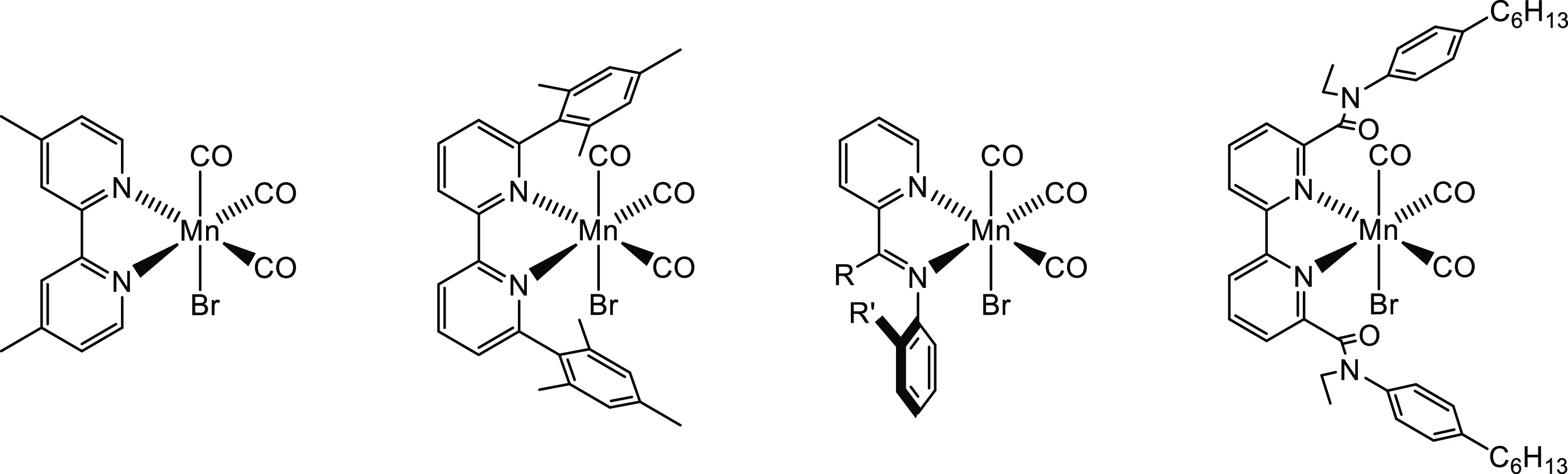
Examples of the previously reported CO_2_ reduction catalysts [MnBr(NN)(CO)_3_] (NN = 4,4′-dimethyl-2,2′-bipyridyl (dmbpy),^9^ 6,6′-bismesityl-2,2′-bipyridyl (mesbpy),^16^ 2-(R-phenyl-R′-imino)pyridine (R_2_-IP),^19^ and 6,6′-bis[*N*-(*p*-hexylphenyl)-*N*-ethyl-amido]-2,2′-bipyridyl (HPEAB)).^20^

Developing methods for efficient light-driven reduction of CO_2_ to industrial feedstocks (CO, HCOOH, MeOH) and solar fuels, such as methane, is a key problem in modern chemistry.^1^ Accordingly, significant efforts have been made to design efficient and selective CO_2_ reduction catalysts.^2,3^ Group 7 metal carbonyl complexes bearing diimine ligands have been studied extensively for this purpose, beginning with the demonstration of photo- and electrochemical catalytic reduction of CO_2_ to CO using [ReCl(NN)(CO)_3_] (NN = diimine ligand, for example, 2,2′-bipyridyl, bpy).^4^ While many catalysts based on the [ReX(NN)(CO)_3_] structure have been developed,^5−8^ the scarcity of extractable Re in the earth’s crust prompted the development of catalysts with earth-abundant metals, such as Mn. The Mn(I) diimine carbonyls of general formula [MnBr(NN)(CO)_3_] have been shown to be effective electrochemical CO_2_ reduction catalysts in the presence of a weak Brønsted acid, such as water.^9,10^ Since 2011, many different Mn(I) complexes have been reported based on this work, which incorporate functionalized polypyridyl ligands (Figure 1).^10−22^ Other chelate ligands have also been used to prepare effective Mn(I) CO_2_ reduction catalysts, such as N-heterocyclic carbenes,^23−28^ phosphinoaminopyridines,^29^ or tridentate NNN, PNP, or CNC “pincer” ligands,^30^ which have been shown to reduce CO_2_ with high turnover frequencies and product selectivity. Recently, a benzothiazole-based Mn(I) complex has been demonstrated to electrochemically reduce CO_2_ in the absence of a proton donor, unlike most other reported Mn(I) catalysts.^31^

An additional advantage of Mn(I) catalysts over their Re(I) counterparts is a reduced required overpotential for electrochemical CO_2_ reduction. This is the case for catalysts that preferentially undergo the “protonation-first” catalytic mechanism rather than the more common, “reduction-first” pathway.^32−35^ It has been demonstrated that addition of proton-donating groups, such as amides or alcohols, to the secondary coordination sphere of the Mn complex can promote the protonation-first pathway.^36,37^

A sustainable and environmentally friendly CO_2_ reduction process would ideally operate in water, and be light-activated. Such systems have been realized for other transition metals, such as Ni, Fe, and Co,^38−42^ but both requirements are difficult to meet for Mn(I) catalysts. Most Mn-based CO_2_ reduction catalysts are only soluble in organic solvents, with the rare example of homogeneous electrocatalytic CO_2_ reduction in aqueous solution enacted by [MnBr(4,4′-dicarboxy-2,2′-bpy)(CO)_3_].^43^ Other studies overcome the lack of water solubility of Mn catalysts by immobilizing them on graphene,^44^ TiO_2_ nanoparticles,^45^ carbon nanotubes, graphitic carbon nitride,^12,22,46,47^ carbon paper or cloth,^48,49^ or polymers.^50^

Light activation of Mn catalysts is precluded by the photosensitivity of typical [MnX(NN)(CO)_3_] compounds, for which excitation even into the lowest energy absorption bands corresponding to the metal-to-ligand (MLCT) or halide-to-ligand (XLCT) charge-transfer transitions results in decomposition through ligand dissociation.^35,51^ Therefore, photosensitizers that absorb light at wavelengths longer than the absorption by the Mn catalyst itself are required to initiate catalysis. To date, photocatalytic CO_2_ reduction using a Mn(I) catalyst has only been demonstrated in organic solvents, utilizing photosensitizers such as [Ru(dmb)_3_]^2+^,^52^ porphyrins, organic dyes, and Cu(I) complexes.^53−56^ Recently, a light-assisted electrocatalytic CO_2_ reduction has been reported, where the Mn–Mn dimer intermediate was prepared electrochemically, and the metal–metal bond was then cleaved by photolysis to form the active catalyst.^57^ One other example of a light-activated Mn(I) catalyst for CO_2_ reduction is a cyanide-bridged Mn dimer that is stable under 395 nm irradiation.^58^ Such advances in photosensitization may also allow for the application of Mn catalysts in photoelectrochemical CO_2_ reduction.^3^

The dissolution of Mn catalysts in water can be achieved by adding a solubilizing functional group of which carboxylates or phosphonates are the most common. These electron-withdrawing groups affect the electronic properties of Mn complexes by decreasing the energy of the lowest unoccupied molecular orbital (LUMO). This also decreases the reduction potential, which is advantageous for electrocatalysis. However, the resultant decrease in the energy of the charge-transfer electronic transition, which shifts the absorption of the Mn(I) complexes into the red region, requires a photosensitizer, which absorbs at even lower energies, to make sure that the photosensitizer can be photoexcited at the wavelengths at which the catalyst does not absorb. Thus, a fine balance between the water solubility and electrochemical and photochemical properties is required in the design of Mn catalysts.^19,59^

**Figure 2:**
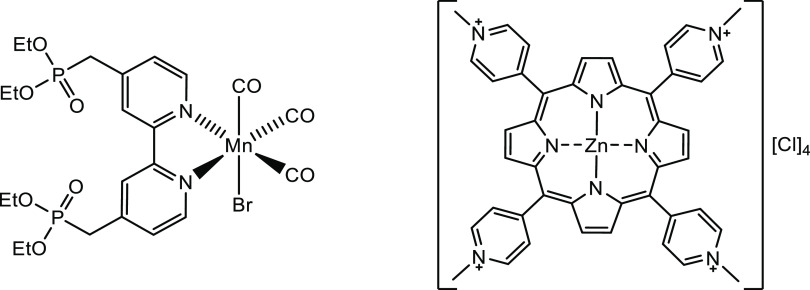
Structures of the catalyst [MnBr(4,4′-{Et_2_PO_3_CH_2_}_2_-2,2′-bipyridyl)(CO)_3_] (**1**) (left) and photosensitizer [ZnTMPyP]Cl_4_ (**2**) (right) used in this study.

To date, no catalytic systems that use Mn(I) catalysts and operate in aqueous solution under photochemical activation with an earth-abundant photosensitizer have been reported. Here, we demonstrate such a system using a Mn(I) catalyst functionalized with pendant phosphonate ester groups, [MnBr(phos-bpy)(CO)_3_] (phos-bpy = 4,4′-{Et_2_PO_3_CH_2_}_2_-2,2′-bipyridyl) (**1**) and a Zn-based photosensitizer (**2**) (Figure 2). To prevent the electron-withdrawing effect of the phosphonate groups from affecting the energy of the charge-transfer transition in **1**, the bipyridyl π-system and the phosphonate group were electronically decoupled by incorporation of a CH_2_-spacer group. We show that the Mn complex (**1**) reduces CO_2_ under 625 nm irradiation when photosensitized by the water-soluble tetra(*N*-methyl-4-pyridyl)porphyrin Zn(II) tetrachloride ([Zn(TMPyP)]Cl_4_) (**2**). Surprisingly, **2** has not been used as a photosensitizer in CO_2_ reduction before. The application of (**1**) as a CO_2_ reduction catalyst has been reported in a recent study that used [Ru(bpy)_3_]^2+^ to photosensitize **1** in dimethylformamide (DMF) under 400 nm light.^13^ The work presented here builds on this previous study to demonstrate the applicability of **1** as a catalyst in water that operates under red light using an earth-abundant photosensitizer. The reactivity of the Mn(I) catalyst toward CO_2_ was investigated by cyclic voltammetry (CV), IR spectroelectrochemistry, and femtosecond transient absorption spectroscopy, with the products analyzed by gas chromatography and multinuclear NMR.

## Experimental Section

Chemical compounds and solvents were purchased from Sigma-Aldrich, Fisher Scientific, and VWR and used as received unless stated otherwise. [NBu_4_][PF_6_] was recrystallized from boiling ethanol prior to electrochemical measurements. Dry solvents were obtained from the University of Sheffield Grubbs solvent purification system. Ar, N_2_, and CO_2_ were supplied by BOC. Tetra(*N*-methyl-4-pyridyl)porphyrin zinc(II) tetrachloride ([ZnTMPyP]Cl_4_) was prepared as described previously.^60^

### Synthesis of [MnBr(4,4′-{Et_2_O_3_PCH_2_}_2_-2,2′-bipyridyl)(CO)_3_], **1**

The synthetic route for the 4,4′-(Et_2_O_3_PCH_2_)_2_-2,2′-bipyridine ligand is detailed in the SI. [MnBr(4,4′-{Et_2_O_3_PCH_2_}_2_-2,2′-bipyridyl)(CO)_3_] was prepared as described previously.^9,12^ Briefly, [Mn(CO)_5_Br] (300 mg, 1.09 mmol) and 4,4′-bis(Et_2_O_3_PCH_2_)-2,2′-bipyridine (465 mg, 1.02 mmol) were dissolved in diethyl ether (50 cm^3^) and then heated to reflux for 4 h in the dark. The reaction vessel was left to cool to room temperature and then further cooled in an ice-water bath to induce precipitation of the product. The precipitate was isolated by vacuum filtration and washed with cold diethyl ether to yield the product as a yellow powder (582 mg, 79%).

υ_max_/cm^–1^ (CH_2_Cl_2_, CaF_2_ cell) 3689 (w, CH), 3601 (vw, CH), 2028 (vs, CO), 1938 (s, CO), 1923 (s, CO), 1620 (w, bpy), 1605 (w, bpy), 1051 (m, PO), 1024 (m, PO); λ_max_/nm (CH_2_Cl_2_) 258 (π–π*), 296 (π–π*), 426 (MLCT); δ_H_ (400 MHz, (CD_3_)_2_SO) 9.13 (d, *J* = 5.5 Hz, 2H, Ar*H*), 8.45 (s, 2H, Ar*H*), 7.64 (s, 2H, Ar*H*), 4.03 (m, 8H, CH_3_C*H*_2_O), 3.59 (d, *J* = 22.6 Hz, 4H, PC*H*_2_), 1.20 (t, *J* = 6.7 Hz, 12H, C*H*_3_CH_2_O); δ_C_{^1^H} (100 MHz, CD_2_Cl_2_) 155.97 (C), 153.67 (CH), 145.64 (*C*), 127.92 (*C*H), 124.33 (*C*H), 63.45 (*C*H_2_), 34.07 (d, *J* = 136.3 Hz, *C*H_2_), 16.77 (*C*H_3_) (not all quaternary carbons were observed); δ_P_{^1^H} (162 MHz, CD_2_Cl_2_) 22.81 (Et_2_O_3_*P*CH_2_); *m*/*z* (TOF MS ES+, Na^+^ added) 457.3 ({Et_2_O_3_PCH_2_}_2_C_10_H_6_N_2_), 479.3 ({Et_2_O_3_PCH_2_}_2_C_10_H_6_N_2_Na), 511.2 (M-3CO–Br), 595.3 (M–Br), 697.2 (M + Na); elemental analysis calcd for C_23_H_30_BrMnN_2_O_9_P_2_: C 40.91%, H 4.48%, Br 11.83%, N 4.15%; found: C 40.18%, H 4.35%, Br 12.36%, N 4.05%. Crystal data for C_23_H_30_BrMnN_2_O_9_P_2_ (*M* = 675.28 g mol^–1^) (CCDC 2119883): triclinic, space group *P*1̅ (no. 2), *a* = 11.2728(5) Å, *b* = 12.1366(6) Å, *c* = 12.4282(6) Å, α = 61.173(2)°, β = 72.144(2)°, γ = 74.311(2)°, *V* = 1402.48(12) Å^3^, *Z* = 2, *T* = 100.0 K, μ(Cu Kα) = 6.333 mm^–1^, *D*_calc_ = 1.599 g cm^–3^, 22 074 reflections measured (1.91° ≤ 2θ ≤ 27.55°), 6307 unique (*R*_int_ = 0.0621), which were used in all calculations. The final *R*_1_ = 0.0500 (*I* > 2σ(*I*)) and w*R*_2_ = 0.0980 (all data).

UV–vis spectroscopy was performed with an Agilent Varian Cary 50 spectrometer. Fourier transform infrared (FTIR) spectroscopy was performed with a PerkinElmer Spectrum One spectrometer with 2 cm^–1^ resolution in a solution cell equipped with CaF_2_ windows.

^1^H and ^13^C NMR spectra were recorded on a Bruker AVIIIHD 400 MHz spectrometer equipped with a 5 mm BBFO SmartProbe. High-resolution mass spectra were recorded using the direct infusion ESI+ TOF method at the University of Sheffield mass spectrometry service. C, H, and N contents were determined using a PerkinElmer 2400 CHNS/O Series II Elemental Analyzer, and the values were accurate to ±0.3%. Gas analysis was performed with a PerkinElmer Autosystem XL gas chromatograph equipped with a thermal conductivity detector (TCD) using He reference gas on a Restek RT-M separation column of a porous layer sieve (5 Å). The column was 30 m long with a diameter of 0.53 mm. One hundred microliters of gas samples were injected directly into the chromatography column. Emission spectroscopy was performed on a Horiba Jobin Yvon Fluoromax 4 spectrofluorometer. Low-temperature emission spectra were recorded in NMR tubes within a custom-made liquid-nitrogen-cooled dewar. Single-crystal X-ray crystallographic diffraction data for [MnBr(4,4′-{Et_2_PO_3_CH_2_}_2_-2,2′-bipyridyl)(CO)_3_] were collected at 100 K by a Bruker D8 Venture diffractometer equipped with a Photon100 CMOS detector using a Cu Kα microfocus X-ray source. Crystals were mounted in fomblin oil on a MiTiGen microloop and cooled in a stream of cold N_2_.

### Cyclic Voltammetry

Cyclic voltammetry was carried out using an Autolab 100 potentiostat and a three-electrode cell with a glassy-carbon working electrode, a Pt-wire counter electrode, and a Ag/AgCl reference electrode. The analyte concentration was 2 × 10^–3^ mol dm^–3^ in a 0.2 mol dm^–3^ solution of the [NBu_4_][PF_6_] supporting electrolyte. The solutions were saturated with N_2_ or CO_2_ prior to performing the measurements. All potentials are quoted relative to the ferrocene/ferrocenium (Fc/Fc^+^) redox couple. The individual redox processes were isolated, and CVs scanned at the rates of 20, 50, 100, 200, and 500 mV s^–1^ to determine electrochemical reversibility. The working electrode was regularly repolished using an alumina–water slurry. Controlled potential electrolysis was carried out in a custom-made glass reaction vessel equipped with a Pt-mesh working electrode, a Pt-wire counter electrode, and an Ag-wire pseudo-reference electrode under CO_2_ atmosphere. Solutions of [NBu_4_][PF_6_] (0.2 mol dm^–3^ in anhydrous CH_3_CN) were used as the electrolyte. The composition of the gas headspace was monitored by gas chromatography. CO concentrations were calculated using a calibration curve constructed with reference gas mixtures made at Sheffield.

### Photocatalytic CO_2_ Reduction Experiments

A solution containing **1** (1.5 × 10^–6^ mol) and **2** (1.9 × 10^–6^ mol) was prepared in deionized H_2_O (2.5 cm^3^) in a 10 mm path length quartz cuvette equipped with a septum seal. Ascorbic acid (25 mg) was then added and the solution purged with CO_2_ for 30 min. The reaction mixture was then stirred and irradiated with 625 nm light supplied by a mounted light-emitting diode (LED) (Thorlabs M625L4, 4 cm^2^ focal area, 308 mW cm^–2^ power density). The focal point of light was set to the center of the quartz cell. The composition of the gas headspace was monitored by gas chromatography. Further details on the gas chromatography method used are provided in the Supporting Information (SI). Experiments were halted after the plateau in CO turnover frequency was reached. Control experiments were carried out under Ar atmosphere to show that CO production was not a result of catalyst decomposition.

### Monitoring of the Reaction Mixture Composition by NMR Spectroscopy

A solution containing **1** (4.9 × 10^–6^ mol), **2** (4.9 × 10^–6^ mol), and ascorbic acid (5 × 10^–4^ mol) was prepared in either D_2_O (5 cm^3^) or D_2_O/H_2_O (90:10 v/v). The reaction mixture was divided into five 1 cm^3^ aliquots in septum-sealed NMR tubes. Tubes 1–4 were purged with CO_2_, while tube 5 was purged with Ar. Then, tubes 1, 2, 3, and 5 were irradiated by the mounted 625 nm LED diode. The composition of the reaction mixture was monitored by ^1^H and ^13^C{^1^H} NMR spectroscopies. The composition of the gas headspace was monitored chromatographically to confirm that catalysis was taking place.

### IR Spectroelectrochemistry

IR-spectroelectrochemistry (IR-SEC) was carried out with an EmStat-3+ Potentiostat. Solutions of the analyte (4 × 10^–3^ mol dm^–3^) with a 0.3 mol dm^–3^ [NBu_4_][PF_6_] supporting electrolyte in anhydrous CH_3_CN were prepared under either Ar or CO_2_ atmosphere. Measurements were performed in an optically transparent thin-layer electrochemical (OTTLE) cell equipped with Pt-mesh working and counter electrodes, an Ag-wire pseudo-reference electrode, and CaF_2_ windows. Spectra were monitored with a PerkinElmer Spectrum One FTIR spectrometer. During IR-SEC, the applied potential was scanned toward negative potential until the onset of the first reduction was reached. At this point, the scan was paused and the spectral changes in the mid-IR region monitored until the first reduction was complete (no further spectral changes observed). The potential scan was then resumed until the second reduction potential was reached, the scan was then paused and the electrolysis monitored until the second reduction process was complete.

### Transient Absorption Spectroscopy

Ultrafast transient absorption spectroscopy was performed at the Lord Porter Laser Laboratory, University of Sheffield. A Ti:Sapphire regenerative amplifier (Spitfire ACE PA-40, Spectra-Physics) provided 800 nm pulses (40 fs full width at half-maximum (FWHM), 10 kHz, 1.2 mJ). Pulses (400 nm) for excitation were generated by doubling a portion of the 800 nm output in a β-barium borate crystal within a commercially available doubler/tripler (TimePlate, Photop Technologies). Excitation pulses (625 nm) were generated from the 800 nm fundamental beam with a commercially available optical parametric amplifier (TOPAS, Light Conversion). White light supercontinuum probe pulses in the range of 440–650 nm were generated in situ using 2% Ti:Sapphire amplifier output, focused on a CaF_2_ crystal. Detection was achieved using a commercial transient absorption spectrometer (Helios, Ultrafast Systems) using a CMOS sensor for the UV–vis spectral range. The relative polarization of the pump and probe pulses was set to the magic angle of 54.7°. Samples were held in 2 mm path length quartz cells and were stirred during experiments. The optical density at the excitation wavelength was ∼0.5. The optical density across the probe range was kept below 1.0.

### Flash Photolysis

Flash photolysis was performed on a home-built setup at the University of Sheffield. A steady-state 150 W Xe arc lamp (Hamamatsu Photonics) was used as the probe source. Sample excitation was achieved with a Nd:YAG laser (LOTIS TII), which provided 355 nm pulses used to pump an optical parametric oscillator (LOTIS TII) to produce 620 nm pulses. Detection was achieved with a Spex Minimate monochromator and FEU0118 PMT. The detector current output was coupled into a Tektronix TDS 3032B digital oscilloscope. The decay traces recorded with the Xe lamp on and off were used to produce the kinetic trace of the decay of the excited state.

## Results and Discussion

### X-ray Crystallography

Crystals for X-ray structure determination were prepared by diffusion of Et_2_O vapor into a solution of **1** in dichloromethane (DCM). The resulting orange block-type crystals were found to be triclinic with the *P*1̅ space group, consistent with previously reported [MnBr(NN)(CO)_3_] complexes (Figure S22). The unit cell contained two molecules of **1**, and no solvent cocrystallized with the complex. The complex formed the expected facial isomer, consistent with previously reported [Mn(X)(L_2_)(CO)_3_] complexes. In the unit cell, the two molecules are offset and rotated 180° from one another, with the axial plane of the Mn center pointed toward the bipyridyl π-system of the other complex, minimizing the steric interaction of the four phosphonate ester groups. No disorder was found within the unit cell of the crystal structure.

To evaluate the effect of functionalization on the single-crystal structure, **1** was compared to the previously reported structure of [MnBr(bpy)(CO)_3_] (CCSD deposition number 977176).^61^ The Mn–N bond lengths and N–Mn–N bite angle were very similar between the two complexes. The C≡O bond was found to be elongated in **1**, which indicates that the electron density on the Mn center is increased, resulting in a larger Mn → CO backdonation, consistent with −CH_2_– spacer exerting some electron-donating effect. This is also shown in the shorter equatorial Mn–C bond in **1** compared to [MnBr(bpy)(CO)_3_]. This increased electron density results from the incorporation of the Et_2_O_3_P–CH_2_– groups and is consistent with the observed slight shifts in the reduction potentials upon functionalization of the bipyridyl ligand with a −CH_2_ group (Table 1).

**Table 1:** Comparison between Selected Bond Lengths and Angles Obtained from the Single-Crystal X-ray Structure of **1** and [MnBr(bpy)(CO)_3_]

parameter	[MnBr(phos-bpy)(CO)_3_]	[MnBr(bpy)(CO)_3_]^61^
Mn–C (axial)	1.812(2) Å	1.803(4) Å
C≡O length (axial)	1.138(3) Å	1.122(5) Å
Mn–C (equatorial)	1.795(4), 1.805(3) Å	1.814(3), 1.809(4) Å
C≡O length (equatorial)	1.148(4), 1.149(5) Å	1.133(5), 1.143(4) Å
M–N length	2.046(3), 2.043(2) Å	2.043(3), 2.052(2) Å
M–Br length	2.159(4) Å	2.5316(10) Å
N–Mn–N bite angle	78.31(9)°	78.59(9)°

### Electronic Absorption Spectra

**Figure 3:**
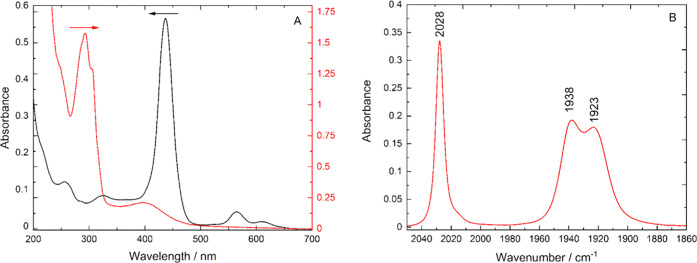
(A) UV–vis absorption spectrum of **1** (red, 3.2 × 10^–5^ mM) and of **2** (black, 1 μM) in H_2_O. (B) IR spectrum of **1** in DCM. Multipeak deconvolutions of the spectra of **1** are given in the SI.

The UV–vis absorption spectrum of **1** in DCM (Figure 3) was similar to previously reported [MnBr(NN)(CO)_3_] catalysts. The absorption bands observed at λ < 350 nm were assigned as bipyridyl-based π–π* transitions. In the 400–550 nm region, a broad absorption envelope with the maximum at 414 nm was observed, which was very similar to [MnBr(dmbpy)(CO)_3_] (Figures S1 and S2). This indicated that the energy of the charge-transfer excited state(s) was not affected by the phosphonate ester groups, confirming that the phosphonate and bipyridyl moieties were electronically decoupled. This observation contrasts with catalysts bearing ring-functionalized bipyridyl ligands in which the introduction of electron-rich or electron-deficient groups affects the energy of the charge-transfer electronic transitions. The deconvolution of the 414 nm peak with pseudo-Voigt functions reveals two overlapping absorption bands assigned to the charge-transfer transitions: a metal–ligand to ligand charge transfer (MLL’CT) from the {Mn(CO)_3_} moiety to the π* orbital of the bipyridyl ligand, and a halide-to-ligand charge-transfer (XLCT) transition.

The absorption spectrum of the porphyrin photosensitizer (Figure 3, black) was typical of symmetric metalloporphyrins. The prominent Soret band attributed to the S_0_ → S_2_ (a_1u_ → e_g_*) transition appeared at 437 nm, and the two Q-bands, corresponding to two S_0_ → S_1_ (a_2u_ → e_g_*) transitions, occurred at 575 and 623 nm.^62,63^

### Infrared Absorption Spectra

The IR absorption spectrum of **1** (Figure 3) in DCM exhibits absorptions typical for [MnBr(NN)(CO)_3_] complexes. The fundamental carbonyl group vibrations were observed in the 2035–1885 cm^–1^ region, which transform with the symmetry species a′(1), a″, and a′(2). A shoulder observed at 2020 cm^–1^ was assigned as a CO/ligand group vibration, consistent with previous observations.^64^ The vibrational frequencies of these four group vibrations are within 5 cm^–1^ of those for [MnBr(dmbpy)(CO)_3_] (Figures S3 and S4), indicating that the electron density on the Mn center is very similar between the two complexes, further confirming that the phosphonate and bipyridyl groups are electronically decoupled. The bands at 1621 and 1635 cm^–1^ were assigned to the stretching vibrations of the bipyridyl rings, and the 1251 cm^–1^ band is attributed to the P=O stretching vibration of the phosphonate ester group.

### Cyclic Voltammetry Data

**Figure 4:**
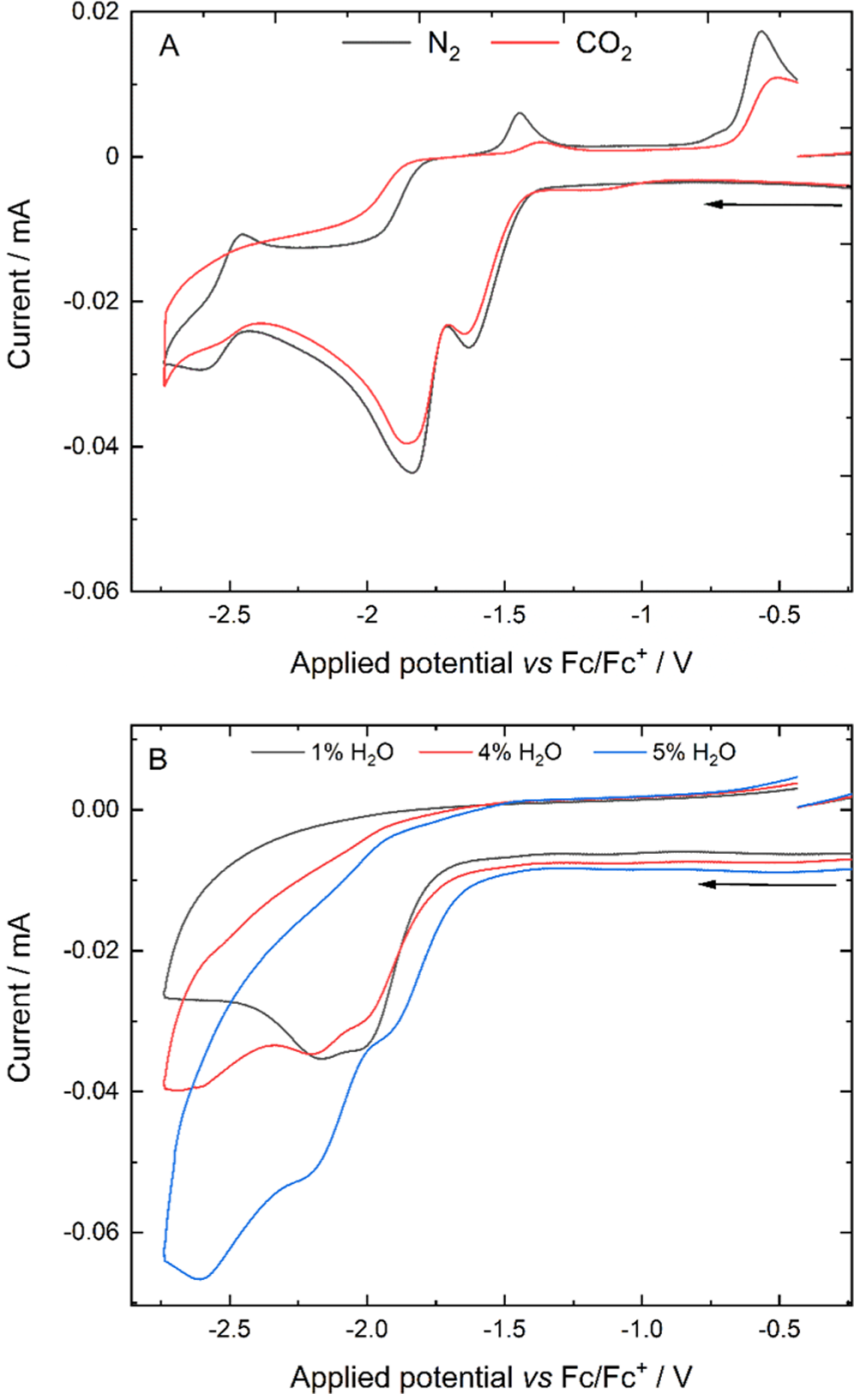
(A) Cyclic voltammograms of a 2 × 10^–3^ mol dm^–3^ solution of **1** and a 0.2 mol dm^–3^ [NBu_4_][PF_6_] electrolyte at a scan rate of 100 mV s^–1^ under N_2_ in anhydrous CH_3_CN (black) and under CO_2_ in anhydrous CH_3_CN (red). (B) Cyclic voltammograms of the CO_2_-purged solution in 1% H_2_O–CH_3_CN (black), 4% H_2_O–CH_3_CN (red), and 5% H_2_O–CH_3_CN (blue).

To determine the ability of **1** to electrochemically reduce CO_2_, a cyclic voltammetry (CV) study was carried out (Figure 4). For a 0.2 mM solution of **1** in CH_3_CN (in the presence of 0.2 M [Bu_4_N][PF_6_] supporting electrolyte) under N_2_ atmosphere, one oxidative and three reductive processes were observed in the range −2.7 to 1.4 V vs Fc/Fc^+^ (Figure 4A). The first and second reduction peaks are the redox processes relevant for CO_2_ reduction. It has been recently shown that the first reduction process corresponds to the reduction of [MnBr(NN)(CO)_3_] leading to [Mn(NN)(CO)_3_]^•^ and Br^–^. The five-coordinate radical is reduced further at this potential to form [Mn(NN)(CO)_3_]^−^, which then reacts with the [MnBr(NN)(CO)_3_] starting material to form [Mn_2_(bpy)_2_(CO)_10_] in a parent–child reduction mechanism. The second reduction peak is associated with reduction of the Mn_2_ dimer to form [MnBr(NN)(CO)_3_]^−^, the active species for electrocatalytic CO_2_ reduction.^65^ The first oxidation and third reduction processes are not utilized in the CO_2_ reductions and hence will not be discussed further. The peak current for the individual redox couples did not linearly depend on the square root of the CV scan rate (Figures S9–S12). Furthermore, repeated CV scans in the full range resulted in permanent changes to the shape of the CV. Thus, the redox processes are only quasi-reversible, as further evidenced by the scan-rate dependent potentials for the redox processes.

The first and second reduction potentials of **1** and [MnBr(dmbpy)(CO)_3_] are very similar and more negative than [MnBr(bpy)(CO)_3_], indicating an increase in the electron density on the bpy functionalized with the −CH_3_ or Et_2_O_3_P–CH_2_ groups. This observation is consistent with the phosphonate group being electronically decoupled, also evident in the UV–vis and FTIR data (Table 2).

**Table 2:** Light Absorption and Electrochemical and Electrocatalytic Properties of [MnBr(NN)(CO)_3_] Complexes in CH_3_CN

catalyst (NN)	MLCT λ_max_/nm	first reduction/V	second reduction/V	Brønsted acid	*E*_cat_, *E*_0_(CO_2_/CO)/V	*i*_cat_/*i*_p_
bpy^9^	416	–1.65^a^	–1.89^a^	nr	nr	nr
dmbpy^9,66^	419	–1.73	–1.98	5% H_2_O	–1.83, −1.29	nr
mesbpy^15,16^	nr	–1.60^a^	nr	0.1 M Mg^2+^	–1.60, −1.40	3.5
				0.3 M TFE	–1.60, −1.40	nr
HPEAB^20^	442	–1.48^a^	–1.74^a^	2% H_2_O	–1.90, −1.40	1.4
				5% H_2_O	–1.90, −1.40	2.3
phos-bpy (**1**)	416	–1.73^a^	–1.86^a^	2% H_2_O	–2.10, −1.40	1.2
				5% H_2_O	–2.10, −1.40	1.9

a Potential given as cathodic peak potential (*E*_p,a_). Reduction potentials were recorded in anhydrous CH_3_CN under an inert atmosphere. Where required, literature data for redox potentials were converted to Fc/Fc^+^ reference with the following conversion constants/mV: Fc/Fc^+^ 0, NHE −630, SHE −624, Ag/AgCl −450, SCE −380, AgNO_3_ −87.^67^ TFE = CF_3_CH_2_OH. nr = data not reported. The *i*_cat_/*i*_p_ values are reported for a scan rate of 100 mV s^–1^.

Under CO_2_ atmosphere, subtle changes to the cyclic voltammogram were observed (Figure 4B), where the reverse peaks for the first and second reduction had lesser peak currents than those observed in the forward scan—again similar to observations made for [MnBr(bpy)(CO)_3_].^9^ This occurred due to the reaction of [Mn(NN)(CO)_3_]^−^ with CO_2_, which prevents the reverse processes from taking place. Under anhydrous conditions, without Brønsted acid, the CO_2_ reduction catalysis could not be initiated, and the CO_2_ reduction process halts after formation of [Mn(NN)(CO)_3_(CO_2_)]^−^, as shown by the IR-spectroelectrochemical data.

Addition of H_2_O to the reaction mixture in the electrochemical cell containing **1** under CO_2_ atmosphere resulted in further changes to the cyclic voltammogram, where the original first and second reduction processes of **1** were no longer observed, and instead, new reduction peaks were found at more negative potentials. These new reduction processes were associated with the CO_2_ reduction catalytic cycle: the first reduction process is associated with initiation of the catalytic reaction and the second occurs at the potential required to ensure turnover of the catalytic cycle. This assignment was evidenced by the increase of the peak current of the second reduction with increasing water concentration under CO_2_ atmosphere. This current enhancement, quantified by the ratio of peak currents under catalytic and inert conditions (*i*_cat_/*i*_p_), reached 1.9 for **1** in a 95:5 CH_3_CN–H_2_O solvent mixture. To confirm that the observed current enhancement was associated with the desired catalysis, a controlled potential electrolysis experiment was used to show that **1** electrocatalytically converts CO_2_ to CO at a potential of −2.3 V vs Fc/Fc^+^ in a 95:5 CH_3_CN/H_2_O mixture.

### Mechanistic Study of Active Catalyst Formation by IR Spectroelectrochemistry

**Figure 5:**
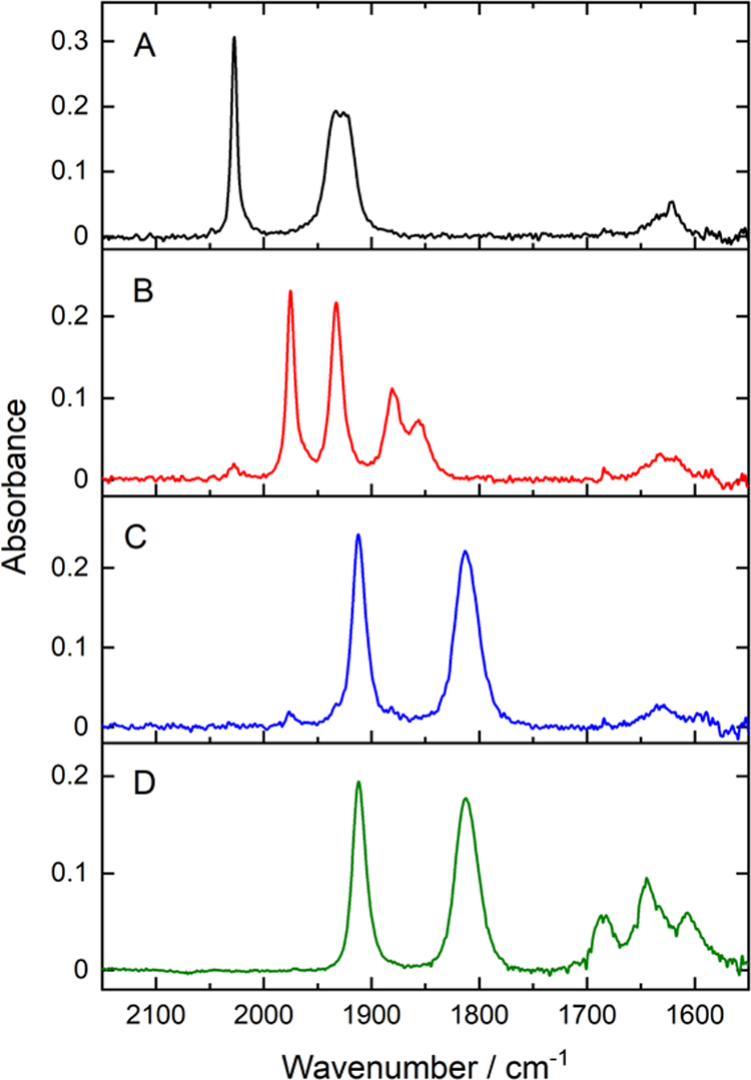
Spectral changes in the 2150–1550 cm^–1^ region of the IR spectrum of **1** following application of the first and second reduction potentials. (A) IR spectrum at 0 V applied potential in Ar-purged anhydrous CH_3_CN. (B) IR spectrum after electrolysis at −1.55 V vs Fc/Fc^+^ in Ar-purged anhydrous CH_3_CN, which corresponds to the first reduction potential. (C) IR spectrum after electrolysis at −2.1 V vs Fc/Fc^+^ in Ar-purged anhydrous CH_3_CN, which corresponds to the second reduction potential. (D) IR spectrum after electrolysis at −2.1 V vs Fc/Fc^+^ in CO_2_-purged anhydrous CH_3_CN, which corresponds to the second reduction potential of **1**.

To further characterize the redox processes observed by CV, the spectral changes following electrochemical reduction were monitored with IR spectroscopy. In **1**, application of the first reduction potential causes a decrease in the intensities of a′(1), a″, and a′(2) CO group absorption bands at 2027, 1935, and 1922 cm^–1^, concurrent with the rise of new absorption bands at 1885, 1880, 1933, and 1975 cm^–1^. These spectral changes were consistent with the commonly reported dissociation of the Br^–^ ligand and subsequent dimerization of the reduced Mn species (Figure 5B).^68−70^ The time resolution of the room-temperature IR-SEC measurement was not sufficient to observe the previously proposed formation of [Mn(NN)(CO)_3_]^•^ or [Mn(NN)(CO)_3_]^−^ at the first reduction potential; the first observable reduction product was the Mn–Mn dimer species.

**Scheme 1:**
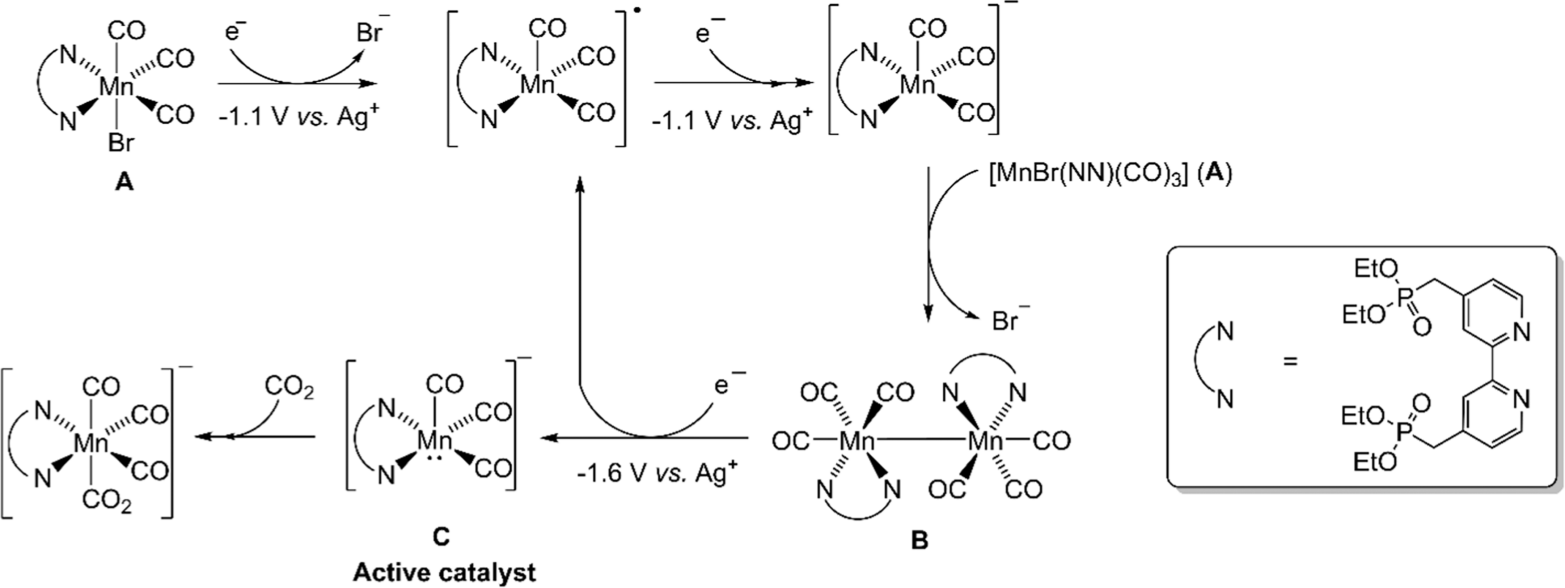
Active Catalyst Formation Mechanism following Two-Electron Reduction of **1** in CO_2_-Purged Anhydrous CH_3_CN, Proposed on the Basis of IR-Spectroelectrochemical Data See Table 3 and Figure 5 for IR absorbances of A–C.

After application of the potential corresponding to the second reduction process, the IR bands associated with the Mn–Mn dimer decay simultaneously with the growth of two bands centered at 1912 and 1812 cm^–1^, which were ascribed to the [Mn(NN)(CO)_3_]^−^ anion (Figure 5C). This species is reported to be the active catalyst that coordinates to CO_2_ to initiate the catalytic cycle.^10,71^ Upon application of a positive potential, the IR absorption bands of the starting complex did not completely reform, consistent with the quasi-reversibility of the reduction processes observed in the CV data. In CO_2_-saturated solution, the same spectral changes were observed during the two-electron reduction process. However, an additional step was now observed, with new bands formed at 1686, 1645, and 1607 cm^–1^. These bands have been previously ascribed to the formation of free monomeric (1685 cm^–1^) and dimeric (1646 cm^–1^) bicarbonates^72^ and formate (1607 cm^–1^) within the electrochemical cell, which are produced as a result of CO_2_ reduction.^19,73^ An additional band attributed to bicarbonate can also be found at 1304 cm^–1^ (Figure S24). The source of the protons required for the electrochemical CO_2_ reduction could be traces of water present in the CO_2_ purge gas. The observed formate is likely produced by a metallohydride intermediate formed by protonation of the active catalyst, and bicarbonate is a known byproduct of CO_2_ reduction in CH_3_CN in the presence of a Brønsted acid. The formation of formate and bicarbonate shows that CO_2_ reduction occurs during electrolysis at −2.1 V vs Fc/Fc^+^, and hence the final spectrum (D) corresponds to the electrocatalytic reaction mixture. Under these conditions, the steady-state concentration of the catalytic intermediates is likely to be too low to be detected by IR absorbances, and the only Mn species that can be discerned is the two-electron reduction product, [Mn(bpy)(CO)_3_]^−^. The proposed catalytic activation mechanism for **1** based on the IR-SEC data and the recent literature is summarized in Scheme 1, and note that the routes for production of formate and bicarbonate CO_2_ reduction products are not shown (Table 3).^65^

**Table 3:** Selective Vibrational Frequencies for Species A–D Observed during the IR-Spectroelectrochemical Study of a Solution of **1** and [NBu_4_][PF_6_] Supporting Electrolyte in Anhydrous CH_3_CN

complex	vibrational frequencies/cm^–1^
[MnBr(phos-bpy)(CO)_3_] (A)	2027, 1935, 1922, 1631, 1625
[Mn(phos-bpy)(CO)_3_]_2_ (B)	1885, 1880, 1933, 1975, 1625, 1631
[Mn(phos-bpy)(CO)_3_]^−^ (C)	1912, 1812, 1631
catalytic reaction mixture (D)	1912, 1812, 1686, 1645, 1607

### Photosensitization of **1** with **2** in Aqueous Solution

**Figure 6:**
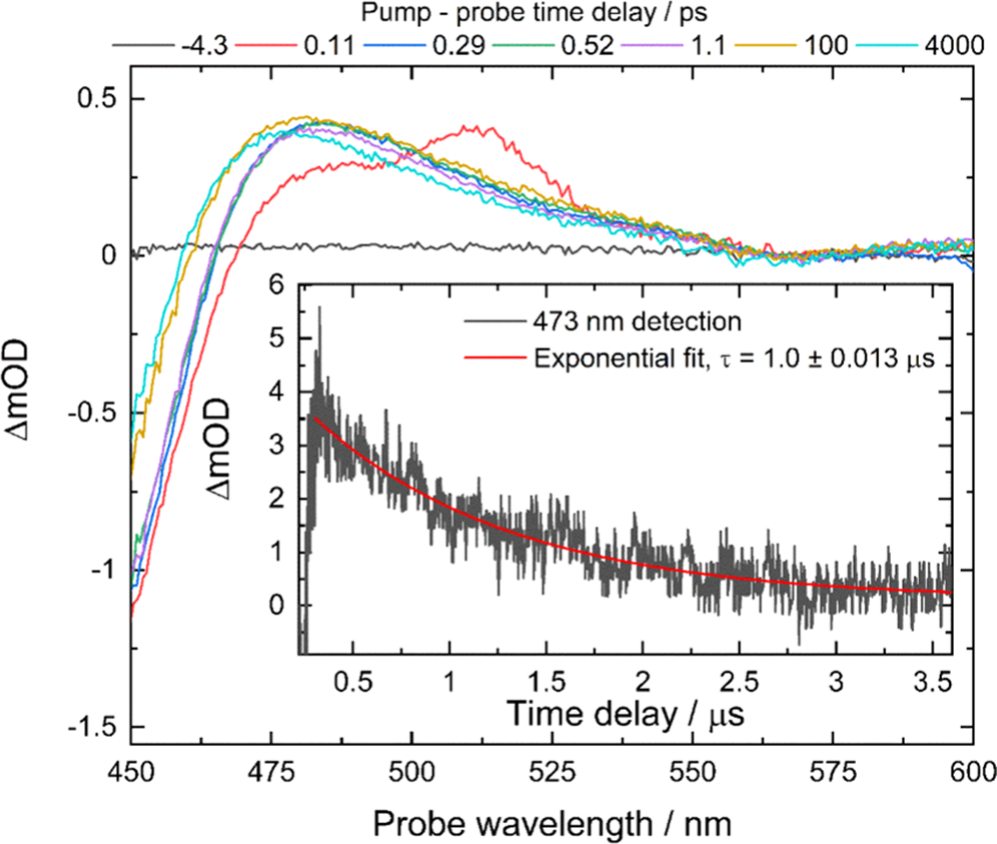
Femtosecond transient absorption spectra of [ZnTMPyP]Cl_4_ (**2**) in H_2_O after 40 fs, 625 nm excitation. Inset: Triplet excited-state decay trace at 473 nm, after 12 ns, 620 nm excitation.

The potential of **1** as a CO_2_ reduction photocatalyst in aqueous solution was investigated using porphyrin **2** as a photosensitizer, and ascorbic acid as the sacrificial electron donor. The porphyrin was chosen for its high water solubility and large extinction coefficient in the 575–650 nm region (Q-bands), where absorption by **1** is negligible (Figure 3). The photophysical properties of **2** in water under red-light irradiation were investigated by time-resolved transient absorption spectroscopy (Figure 6) using excitation with a 40 fs, 625 nm laser pulse and a broad-band probe in the range 420–600 nm. At very small time-delays (*t* < 150 fs), two transient absorption bands were observed at 511 and 485 nm, corresponding to the singlet and triplet excited states of **2**. The spectral profile was consistent with previously reported data following excitation of **2** at 404 nm.^74^ By 500 fs, the 511 nm band decays, indicating that intersystem crossing (ISC) from S_1_ to T_1_ was complete. Due to convolution with the instrument response time, it was not possible to obtain an exact time-constant for the ISC. After 500 fs, no further spectral changes occur up to the longest available time delay of 7 ns. The lifetime of the T_1_ state of **2** in aerated water, measured by microsecond flash photolysis following 600 nm, ∼12 ns excitation, was found to be 1.0 (±0.013) μs and is sufficiently long to allow diffusion-controlled electron transfer to take place. Hence, **2** was used to photoreduce **1**, initiating CO_2_ reduction.

**Figure 7:**
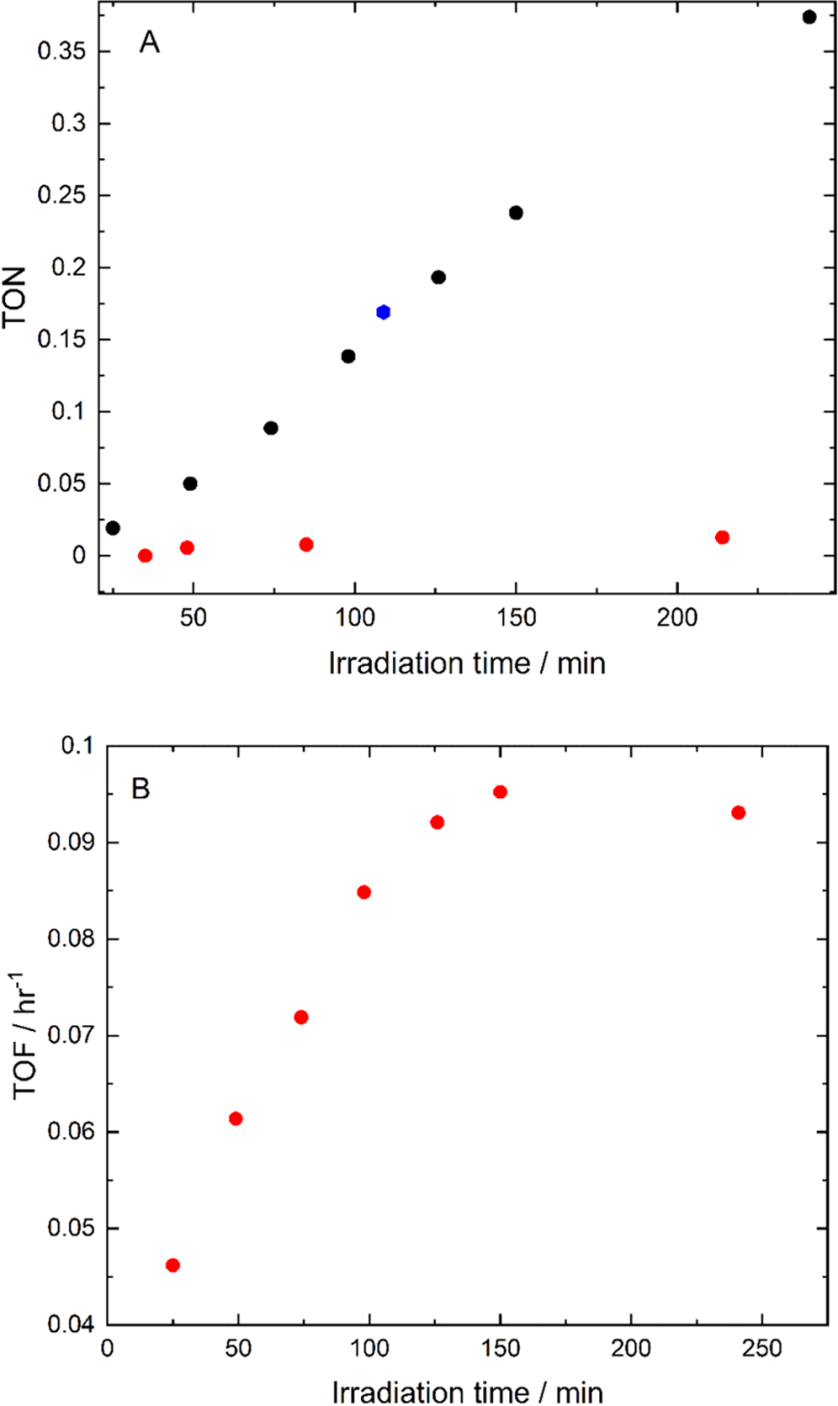
(A) Turnover number vs irradiation time with 625 nm [308 mW cm^–2^] for the first cycle of the catalytic reaction (black). The TON value following repurging of the reaction mixture with CO_2_ is shown in blue. Control experiment data without photosensitizer **2** shown in red. (B) Turnover frequency against irradiation time for the catalytic reaction.

Irradiation of an aqueous solution of **1** and **2** in the presence of ascorbic acid with 625 nm light resulted in the evolution of CO gas bubbles from the liquid phase. The rate of CO formation was monitored by gas chromatography and quantified with the turnover number (TON) and turnover frequency (TOF) (Figure 7). CO was produced at a continuous rate during the catalytic experiment. The photochemical TOF_max_ was reached after 150 min of irradiation. To assess catalyst recyclability, the reaction mixture was repurged with CO_2_ after 250 min of irradiation and then irradiated for a further 110 min. The catalytic performance in this second cycle was nearly identical to the first cycle, indicating that catalyst degradation was not a significant problem. A control experiment, carried out in the absence of CO_2_, resulted in negligible CO formation (Figure S14), confirming that the CO observed was produced by CO_2_ reduction and not through decomposition of the catalyst. The TOF of CO production for this catalytic system was significantly lower than previously reported examples of photochemical CO_2_ reduction with Mn(I) catalysts. The apparent quantum yield for the photocatalysis, estimated by taking the ratio of the rate of CO formation and the number of photons incident on the reaction mixture per hour, was 2.67% for the initial 25 min irradiation period and decreased over time to a value of 1.32% at 4 h (see SI). The decrease in quantum yield over time was attributed to the photodecomposition of **2**, which was observed by NMR spectroscopy (see below).

The CO_2_ reduction cycle involves two protonation steps; thus, the pH of the reaction mixture can have a large influence on the rate of catalysis. The initial pH of the reaction mixture of **1**, **2**, and ascorbic acid in CO_2_-purged water was 4.5. The pH increased to 5.0 after irradiation of the mixture with 625 nm light for 2.5 h, suggesting that the CO_2_ and ascorbic acid within solution had been partially depleted. In acidic solution, it is possible that proton reduction by the Mn(I) catalyst becomes competitive with CO_2_ reduction. However, observable levels of H_2_ were not detected within the reaction mixture headspace by gas chromatography.

Rough estimation of the Gibbs energy of electron transfer was performed using Weller’s formula (Δ*G*_et_ = *E*_ox_ – *E*_red_ – *E*_00_), where *E*_00_ is the transition energy between the lowest vibrational levels of the excited and ground states of the photosensitizer. This value was estimated from the emission spectrum of **2** recorded at 77 K (Figure S23), where the lowest energy emission band was found at 671 nm (1.85 eV). The reported oxidation potential for **2** is 1.81 V vs Fc/Fc^+^.^75^ Taking into account the reduction potentials for **1** (Table 2), one finds Δ*G*_et_ = 1.7 and 1.8 V for the first and second reductions of **1** by the photoexcited porphyrin, respectively. As Δ*G*_et_ is significantly positive, the required electron transfer is thermodynamically unfavorable, which is the likely origin of the very slow rate of CO_2_ reduction observed during photocatalysis.

A reductive quenching mechanism is also possible, where ascorbic acid can quench the porphyrin excited state to produce a one-electron reduced porphyrin (*E*_red_ = −0.85 V,^76^ Δ*G*_et_ = −0.53 V). However, the required electron transfer from the one-electron reduced porphyrin to **1** is also thermodynamically unfavorable (Δ*G*_et_ = 1.95 V).

### Reaction Monitoring by NMR Spectroscopy

Changes in the reaction mixture composition during catalysis were monitored by NMR spectroscopy. A solution of **1**, **2**, and ascorbic acid was prepared in D_2_O under CO_2_ atmosphere. Prior to irradiation, six ^1^H resonances were observed in the 7.5–9.2 ppm region of the spectrum. These were assigned to either the porphyrin (9.13, 8.95, 8.81 ppm) or the Mn catalyst (9.11, 8.24, 7.56 ppm) (Figures S15 and S16). Irradiation of the reaction mixture with 625 nm light for 16 h resulted in significant changes to the NMR spectrum but did not result in complete conversion of **1** to the active catalyst.

The partial conversion of **1** to the active catalytic species was evidenced by a decrease in intensity of the ^1^H resonances during the catalytic experiment, concomitant with the formation of new resonances at 7.71, 8.27, and 8.57 ppm. This was tentatively ascribed to the reaction of **1** to form intermediate catalytic species, such as the Mn–Mn dimer and active catalyst. The slow rate of formation of these intermediates from **1** was thought to result from inefficient photosensitization by **2**, in agreement with the small TOF observed during catalysis and low apparent quantum yield of CO formation (Figure 7). The ^1^H resonances of the remaining [MnBr(NN)(CO)_3_] starting material did not undergo any changes in multiplicity or chemical shift over the 16 h irradiation period, which demonstrates the stability of **1** under 625 nm irradiation prior to its reduction by the photosensitizer.

The proton resonances of **2** were found to decay to the spectral baseline within 1 h of irradiation, indicating that the porphyrin was permanently changed during catalysis. A new resonance observed at 8.64 ppm was assigned to the resulting photoreduced porphyrin species, as evidenced by a control experiment, where **2** was irradiated with 625 nm light in the presence of ascorbic acid (Figure S20). The UV–vis absorption spectrum of the NMR sample recorded before and after this irradiation period revealed that the porphyrin decomposition product had a new absorption band at lower energies compared to the Q-bands of **2** (Figure S21). This was consistent with the formation of a chlorin species, a known photoreduction pathway for metalloporphyrin complexes.^77,78^ The rate of porphyrin photoreduction was significantly reduced in the absence of **1**, and the photoreduction product is seemingly stable under 625 nm irradiation. It is unknown whether the porphyrin reduction product was able to photosensitize the Mn catalyst; thus, the degradation of the photosensitizer may be an additional factor in the observed low catalytic performance.

Two further control experiments were carried out to confirm the NMR spectral assignments. First, a solution of **1**, **2**, and ascorbic acid irradiated with 625 nm light under argon atmosphere resulted in the formation of new ^1^H resonances at 7.71, 8.27, 8.57, and 8.64 ppm (Figure S15). The NMR spectrum recorded under these conditions was effectively the same as that observed under CO_2_ atmosphere, which supports assignment of the new resonances as (i) a photoreduced porphyrin species and (ii) intermediate Mn complexes, such as the Mn–Mn dimer and active catalyst. Notably, we do not observe resonances attributed to intermediates of the CO_2_ reduction catalytic cycle under CO_2_ atmosphere, which likely results from the low steady-state concentration of these species.

The second control experiment monitored the solution of **1**, **2**, and ascorbic acid, under CO_2_ but without light. Here, no changes in the NMR spectrum were observed during the 16 h experiment, confirming the stability of **1** and **2** in D_2_O solution in the dark.

The catalytic studies were repeated in a 9:1 H_2_O–D_2_O mixture, where the resulting NMR spectra were similar to those observed in pure D_2_O (Figures S17 and S18). Again, the formation of a photoreduced porphyrin species was evidenced by the new resonance at 8.6 ppm. In H_2_O–D_2_O, the resonances of the intermediate Mn species at 7.71 and 8.57 ppm were much broader and weaker than in pure D_2_O. It is tentatively suggested that the additional broadening observed was a result of a slightly faster catalytic turnover, which decreased the effective steady-state concentration of the active catalyst, consistent with previous observations of a H/D kinetic isotope effect in Mn-catalyzed CO_2_ reduction.^36^ No evidence of (CO_2_H)^−^, which has an expected chemical shift in H_2_O–D_2_O of 8.45 ppm, was found in the ^1^H NMR spectra. To confirm that formate was not produced, an experiment was carried out where the reaction mixture was purged with isotopically labeled ^13^CO_2_. The ^13^C{^1^H} NMR spectra of the reaction mixture in the presence of ^12^CO_2_ or ^13^CO_2_ were found to be identical except for the ^13^CO_2_ resonance at 125 ppm (Figure S19). Together, these NMR experiments show that CO_2_ reduction with **1** in water results in selective formation of CO as the CO_2_ reduction product when photosensitized by **2**.

## Conclusions

A water-soluble Mn(I) diimine complex [MnBr(4,4′-{Et_2_O_3_PCH_2_}_2_-2,2′-bipyridyl)(CO)_3_] has been shown to catalyze the reduction of CO_2_ to CO both electrochemically and photochemically under red-light irradiation in aqueous solution. The incorporation of {−P(O)(OEt)_2_} groups, decoupled from bpy by a −CH_2_– spacer, achieved water solubility of **1** without modifying the electronic properties of the catalyst. Consistent with other [MnBr(NN)(CO)_3_] catalysts, IR-spectroelectrochemical studies show that the electrochemical reduction of CO_2_ with **1** proceeds via a five-coordinate anion [Mn(phos-bpy)(CO)_3_]^−^ formed in a multielectron reduction process through the usual Mn–Mn dimer intermediate. For the first time, it is shown that a manganese complex **1** reduces CO_2_ to CO in water under red-light (625 nm) irradiation using a [ZnTMPyP]^4+^ photosensitizer with no production of formate. Further, after the TOF in the photochemical CO_2_ reduction had reached a plateau, repurging the reaction mixture with CO_2_ restarted catalysis at its original rate, demonstrating the recyclability of **1**. Despite **1** photodecomposing rapidly under <500 nm light, the photostability of the active catalyst under catalytic conditions was confirmed by ^1^H and ^13^C{^1^H} NMR spectroscopy over at least 16 h of red-light irradiation. The slow rate of photocatalytic CO_2_ reduction was ascribed to inefficient electron transfer from the porphyrin to the catalyst, which results from the low triplet excited-state energy of the metalloporphyrin.

Overall, this work demonstrates an example of noble-metal-free photocatalytic CO_2_ reduction in water with a Mn molecular catalyst. Complex **1** presents a promising platform for further development of earth-abundant CO_2_ reduction catalysis, where replacement of the Zn-porphyrin with a more effective photosensitizer could unlock the potential of the intrinsically photosensitive Mn(I)-diimine catalyst for CO_2_ reduction in aqueous solution, using red light.

## Supporting Information Available

Further experimental information, synthetic methods, and additional NMR, FTIR, UV–vis, emission spectroscopy, and catalytic and electrochemical data (PDF)
