# Supplementary material for: Photocatalytic Reduction of CO2 to CO in Aqueous Solution under Red-Light Irradiation by a Zn-Porphyrin-Sensitized Mn(I) Catalyst
Source: Inorg Chem. 2022 Aug 12;61(34):13281–92. doi: 10.1021/acs.inorgchem.2c00091 (PMC9446891; doi:10.1021/acs.inorgchem.2c00091)
Supplement: Supplementary file 1 — ic2c00091_si_001.pdf [file ic2c00091_si_001.pdf]

Supporting information

Photocatalytic reduction of CO_2 to CO in aqueous solution under red light irradiation by a Zn-porphyrin-sensitized Mn(I) catalyst

James Shipp,[a] Simon Parker,[a] Steven Spall,[a] Samantha L. Peralta-Arriaga, Craig C. Robertson,[a] Dimitri Chekulaev,[a] Peter Portius,[a] Simon Turega,[b] Alastair Buckley,[c] Rachael Rothman,[d] and Julia A. Weinstein[a*]

a) Department of Chemistry, University of Sheffield, S3 7HF, UK

b) Department of Chemistry, Sheffield Hallam University, S1 1WB, UK

c) Department of Physics and Astronomy, University of Sheffield, S3 7RH, UK

c) Department of Chemical and Biological Engineering, University of Sheffield, S1 3JD, UK

Part 1: Materials and Methods

All preparative procedures were carried out using standard techniques. All commercially available reagents were laboratory grade and used as received unless otherwise stated. Dry solvents were obtained from the Grubbs dry solvent service at the University of Sheffield. Air sensitive reactions were carried out under argon delivered through a vacuum-gas manifold at a relative pressure of 0.1 bar. Vacuum was established with a mineral oil sealed rotary vane pump, the ultimate vacuum achievable was $8x10^{-2}$ mbar.

Gas chromatography

Detection of gaseous products was done using gas chromatography. The gas analysis was performed with a Perkin-Elmer Autosystem XL gas chromatograph equipped with a thermal conductivity detector (TCD). The TCD compared the thermal conductivities of the reference gas (He) with the carrier gas (H_2 for CO detection, N_2 for hydrogen detection) and analyte. The separation column was a Restek RT-M porous layer sieve (5 Å). The column was 30 m long with a diameter of 0.53 mm. The chromatograph method file was designed to achieve maximum separation between the carbon monoxide products and trace nitrogen remaining in the samples. The method file was set-up with the following parameters:

Injection volume: 100 μL

Starting temperature: 30 °C

TCD temperature: 100 °C

Carrier gas: hydrogen

Carrier gas flow rate: 5 cm^3 min^{-1}

Temperature ramp: Hold at 30 °C for three minutes. Then increase the oven temperature by 10 °C min^{-1} until the oven reaches 100 °C. Hold at 100 °C for one minute.

Preparation of tetra(N-methyl-4-pyridyl)porphyrin zinc (II) tetrachloride

This complex was prepared according to a literature procedure.[1]

Preparation of dimethylphenylbenzimidazoline (BIH)

This compound was prepared according to a literature procedure.[2]

Preparation of 4,4'-bis(diethoxymethylphosphonato)-2,2'-bipyridine

This ligand was prepared according to a modified literature procedure.[3]

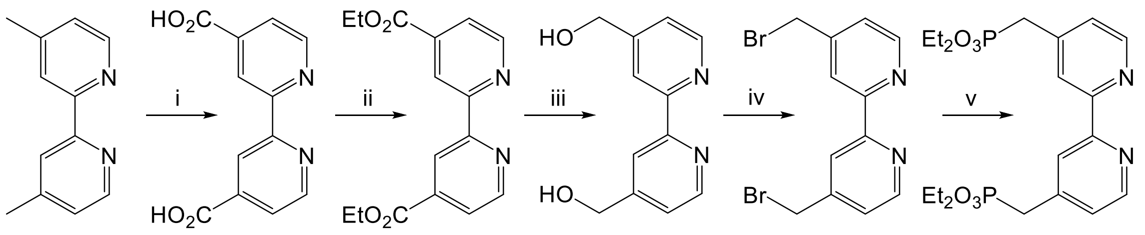

i) $K_2Cr_2O_7/H_2SO_4$ (85%), ii) EtOH-H_2SO_4 (90%), iii) $NaBH_4$-EtOH (81%),
iv) HBr-H_2SO_4 (85%), v) $P(OEt)_3$ (80%)

Scheme S1 – Reaction scheme for the preparation of 4,4'-bis(diethoxymethylphosphonato)-2,2'-bipyridine.

4,4'-dicarboxy-2,2′-bipyridine

4,4′-dimethyl-2,2′-bipyridine (2.5 g 13.5 mmol) was dissolved in of 98% H_2SO_4 (100 cm^{-3}). To this solution, potassium dichromate (12 g) was added in portions whilst stirring, ensuring the temperature remained between 40-80 °C. The reaction mixture was then cooled in an ice bath, and the resulting precipitate isolated by vacuum filtration. The precipitate was then refluxed for 16 h in 50% HNO_3 (50 cm^3), then poured over ice and subsequently diluted to with water (400 cm^3) and left to warm to room temperature. The resulting white precipitate was then filtered off, washed with water and acetone and dried under a vacuum to yield the product (2.29 g, 91%).

^{1}H NMR (400 MHz, d^6-DMSO) δ 8.93 (dd, J = 4.9, 0.7 Hz, 2H), 8.87 – 8.83 (m, 2H), 7.92 (dd, J = 4.9, 1.6 Hz, 2H).

4,4′-bis(ethoxyester)-2,2′-bipyridine

4,4′-dicarboxy-2,2′-bipyridine (1.05 g, 4.12 mmol) was suspended in ethanol (40 cm^3) then 98% H_2SO_4 (2 cm^3) was added. The mixture was refluxed at 85 °C under inert atmosphere for 24 hours to give a pale pink solution. Removal of the solvent under reduced pressure left a pale pink oil, which was mixed with water (20 cm^3) and extracted with chloroform (3 x 50 cm^3). The combined organic fractions were dried over anhydrous $MgSO_4$. The mixture was then filtered, and the volume reduced under reduced pressure to approximately 20 cm^3. Addition of methanol (20 cm^3) resulted in formation of a pale pink precipitate, which was isolated by vacuum filtration and then dried under high vacuum to yield the product (964 mg, 76%).

^{1}H NMR (400 MHz, $CDCl_3$) δ 8.97 (s, 2H), 8.89 (d, *J* = 4.9 Hz, 2H), 7.94 (dd, *J* = 5.0, 1.6 Hz, 2H), 4.48 (q, *J* = 7.1 Hz, 4H), 1.47 (t, *J* = 9.2, 5.0 Hz, 6H).

4,4′-bis(hydroxymethyl)-2,2′-bipyridine

4,4′-bis(ethoxyester)-2,2′-bipyridine (750 mg, 2.4 mmol) was suspended in ethanol (50 cm^3) followed by the addition of sodium borohydride (2 g, 53 mmol). The mixture was refluxed at 65 °C under inert atmosphere for 3 hours. A gel was observed forming on the surface of the reaction mixture after approximately 1 hour, which was dissolved by addition of additional ethanol (25 cm^3). After cooling to room temperature, a saturated aqueous solution of NH_4Cl (100 cm^3) was added to the mixture and stirred for 15 min. The ethanol was removed under reduced pressure and the resulting white solid was dissolved in the minimum volume of water (*ca.* 150 cm^3). The solution was extracted with ethyl acetate (5 x 50 cm^3), then the combined organic fractions were dried over anhydrous $MgSO_4$. The solvent was removed under reduced pressure, yielding the product as a pale pink solid (306 mg, 50%).

^{1}H NMR (400 MHz, d^6-DMSO) δ 8.60 (δ, *J* = 4.9 Hz, 2H), 8.39 (s, 2H), 7.40 – 7.34 (m, 2H), 5.56 (t, *J* = 5.8 Hz, 2H), 4.63 (d, *J* = 5.8 Hz, 4H).

4,4′-bis(bromomethyl)-2,2′-bipyridine

4,4′-bis(hydroxymethyl)-2,2′-bipyridine (674 mg, 3.12 mmol) was dissolved in 48% HBr (40 cm^3) and 98% H_2SO_4 (14 cm^3). The resulting fuming orange solution was refluxed at 100 °C for 18 hours. Upon cooling to room temperature, the mixture was neutralized with a saturated aqueous solution of NaOH (*ca.* 200 cm^3). The resulting white precipitate was isolated by vacuum filtration, washed with water (300 cm^3), and then left to dry. The solid was then dissolved in

chloroform (40 cm^3), dried over anhydrous $MgSO_4$ and filtered. The solvent was removed under reduced pressure, yielding the product as a white solid (842 mg, 79%).

^{1}H NMR (400 MHz, $CDCl_3$) δ 8.70 (d, *J* = 5.0 Hz, 2H), 8.46 (d, *J* = 1.2 Hz, 2H), 7.39 (dd, *J* = 5.0, 1.8 Hz, 2H), 4.51 (s, 4H).

4,4′-bis($Et_2O_3PCH_2$)-2,2′-bipyridine

A solution of diethyl phosphite (15 cm^3) in chloroform (10 cm^3) was purged with N_2 for 30 min. This mixture was transferred to a flask containing 4,4′-bis(bromomethyl)-2,2′-bipyridine (842 mg, 2.46 mmol) and subsequently refluxed at 85 °C for 6 hours. The reaction mixture was allowed to cool to room temperature, and then the solvent and excess diethyl phosphite were removed under reduced pressure to yield the crude product as a pale pink solid. The crude product was purified by column chromatography (SiO_2, 80:20 ethyl acetate: methanol) to yield the purified product as a pale yellow oil. The oil was left overnight, resulting in crystallisation of the product as an off-white solid (942 mg, 64%).

^{1}H NMR (250 MHz, $CDCl_3$) δ 8.60 (d, *J* = 5.0 Hz, 2H), 8.33 (s, 2H), 7.42 – 7.28 (m, 2H), 4.07 (dq, *J* = 8.1, 7.1 Hz, 8H), 3.23 (d, *J* = 22.2 Hz, 4H), 1.27 (t, *J* = 7.1 Hz, 12H). ^{31}P NMR (101 MHz, $CDCl_3$) δ 24.30 (s). MS (ESI, +ve): *m/z* 457.2 (MH^+).

Part 2: Additional characterisation data for [Mn({$Et_2O_3PCH_2$}$_2$bpy)(CO)$_3$(Br)]

UV-vis spectroscopy

Visible absorption spectroscopy of the manganese complex shows successful coordination of the bipyridyl ligand to the complex, as evidenced by the π–π* and MLCT electronic absorption bands. The UV-vis spectrum showed that it would be possible to irradiate the CO_2 reducing systems at 625 nm with no light absorption from the catalyst. Therefore, photolysis could be prevented through the use of porphyrins as photosensitisers.

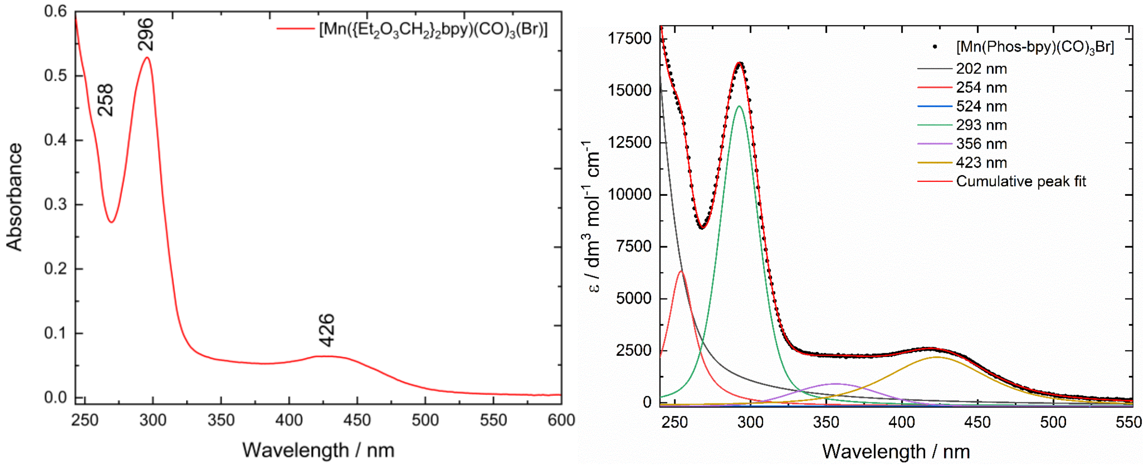

Figure S1 – UV-vis absorption spectra of [Mn(phos-bpy)(CO)$_3$Br] in dichloromethane, the pseudo-Voigt profiles used to deconvolute the absorption spectrum are shown on the right.

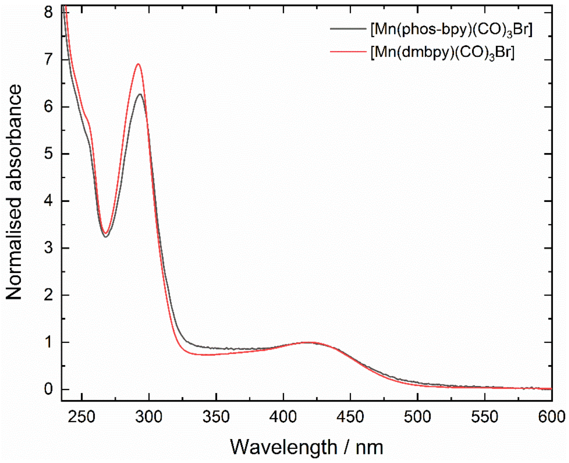

Figure S2 – UV-vis spectra of [Mn(phos-bpy)(CO)$_3$Br] and [Mn(dmbpy)(CO)$_3$Br] in dichloromethane, normalised to the MLCT/XLCT maxima.

FT-IR spectroscopy

FT-IR spectra of the analyte complex in DCM provide evidence for the proposed structure. It was possible to identify vibrational modes attributed to the CO, CH and PO bond vibrations.

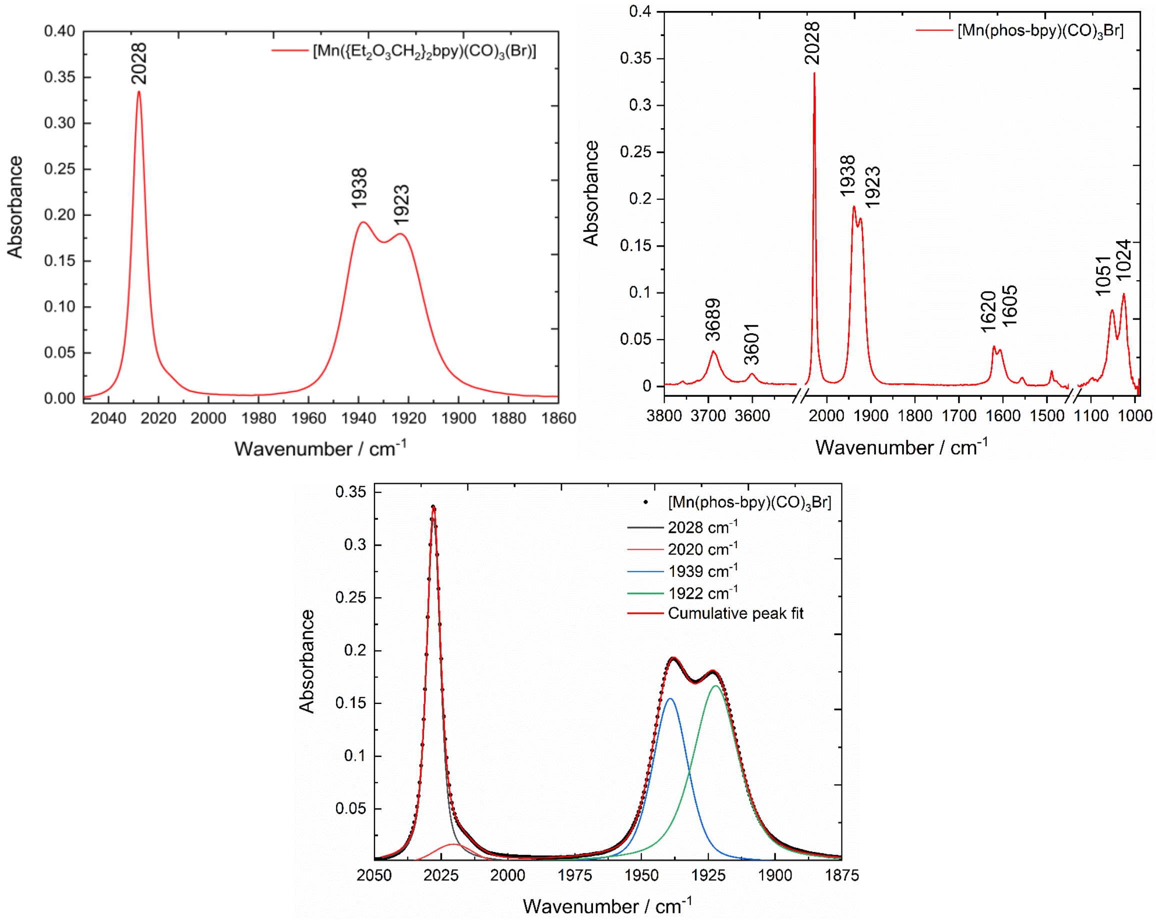

Figure S3 – IR absorption spectra of [Mn(phos-bpy)(CO)$_3$Br] in dichloromethane, the pseudo-Voigt profiles used to deconvolute the carbonyl group vibrations are shown at the bottom.

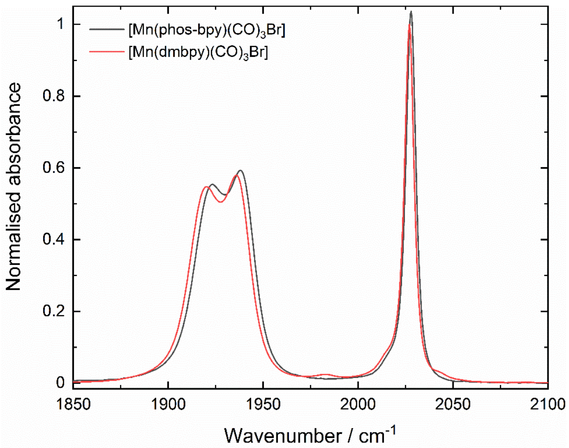

Figure S4 – IR spectra of [Mn(phos-bpy)(CO)$_3$Br] and [Mn(dmbpy)(CO)$_3$Br] in dichloromethane, normalised to the a'(1) maxima.

^{1}H NMR:

^{1}H NMR was consistent with the proposed structure, showing three resonances for the bipyridyl environments, and three alkyl environments for the methylene and ethyl moieties. Note that rapid catalyst photolysis within the NMR sample whilst queued can adversely affect the ^{1}NMR spectrum, resulting in the formation of weak resonances corresponding to the photolysis products.

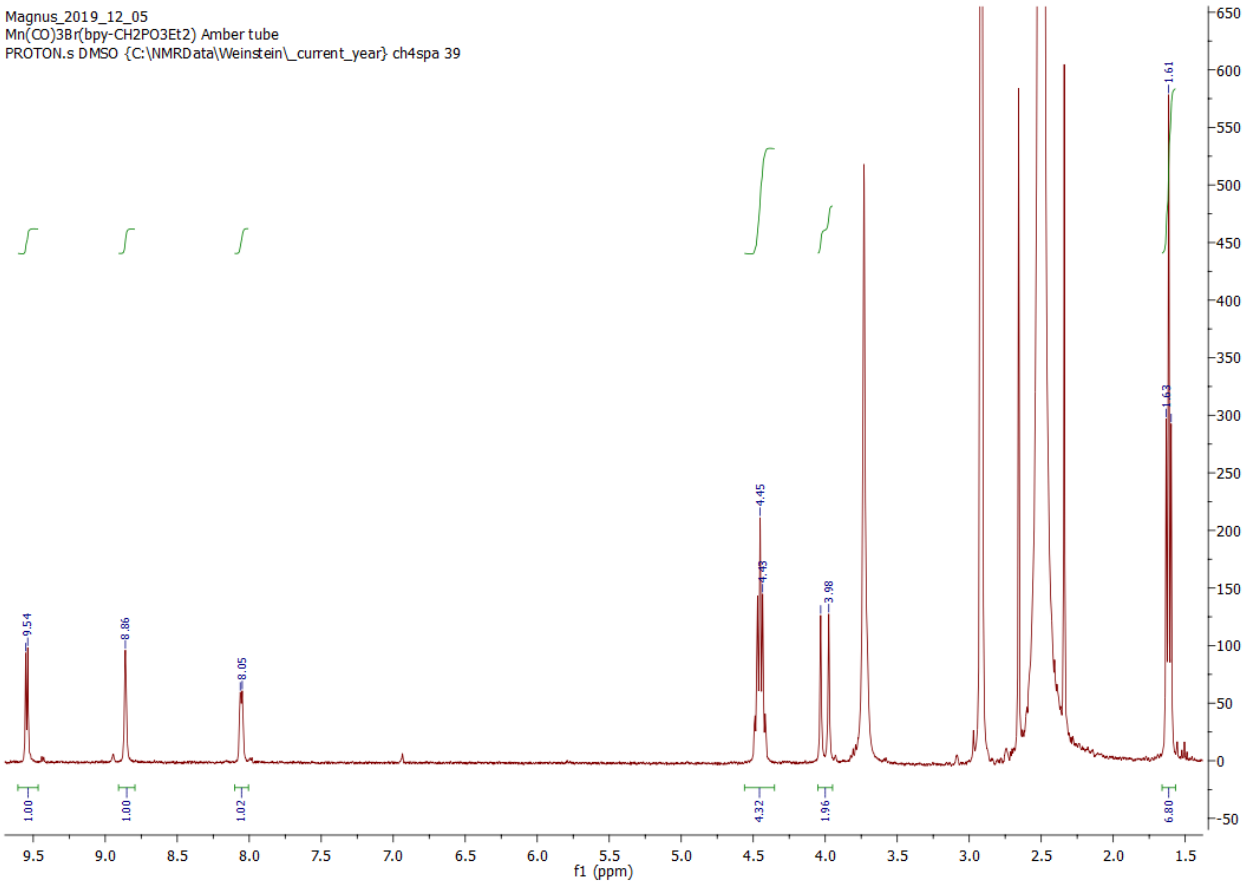

Figure S5 – ^{1}H NMR spectrum of [Mn(phos-bpy)(CO)$_3$Br] in d^6-dimethylsulphoxide.

^{13}C NMR:

The methylene group in this complex appears as a doublet due to the coupling between the ^{31}P and ^{13}C nuclei. The small peak at δ=31 is the *C*H$_3$ resonance of acetone. All other signals are attributed to the analyte complex, not all quaternary carbons were observed.

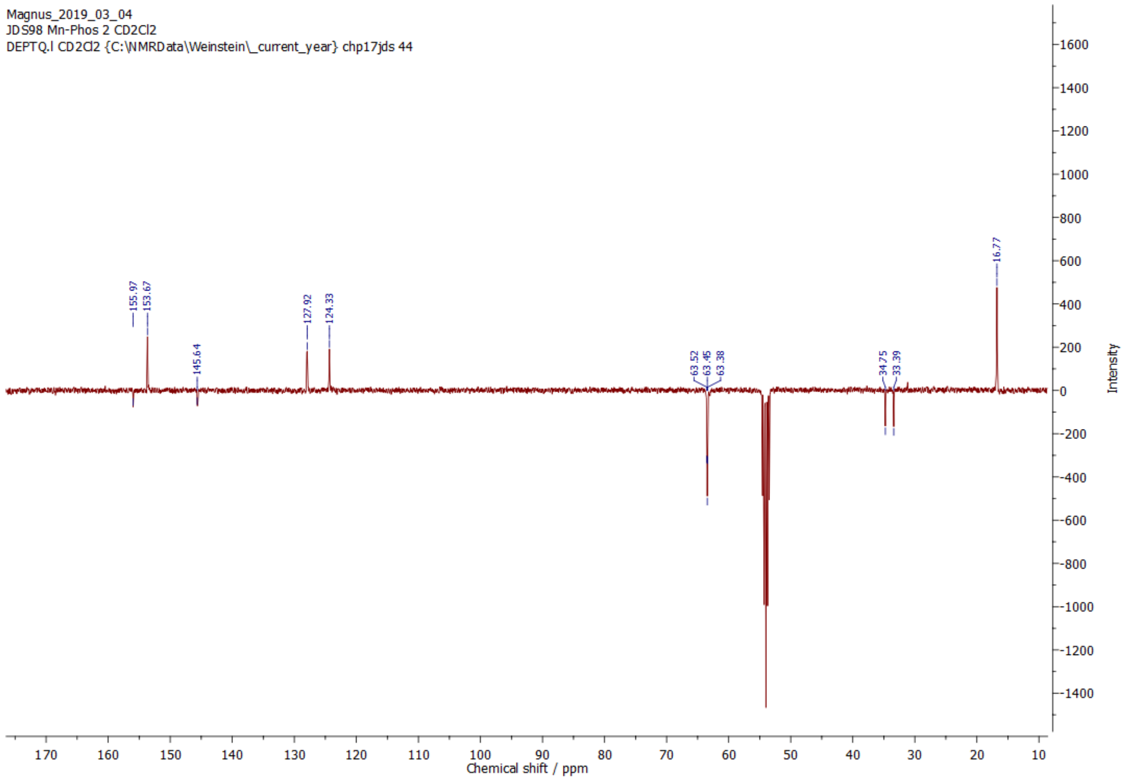

Figure S6 – $^{13}C\{^{1}H\}$ NMR spectrum of [Mn(phos-bpy)(CO)$_3$Br] in d^2-dichloromethane.

^{31}P NMR

A single resonance was observed in the proton decoupled ^{31}P spectrum. The resonance appears to be a singlet with a small shoulder. This was likely due to convolution with a doublet resulting from ^{31}P-^{13}C coupling. The doublet is much less intense than the singlet peak due to the low abundance of ^{13}C compared to ^{31}P.

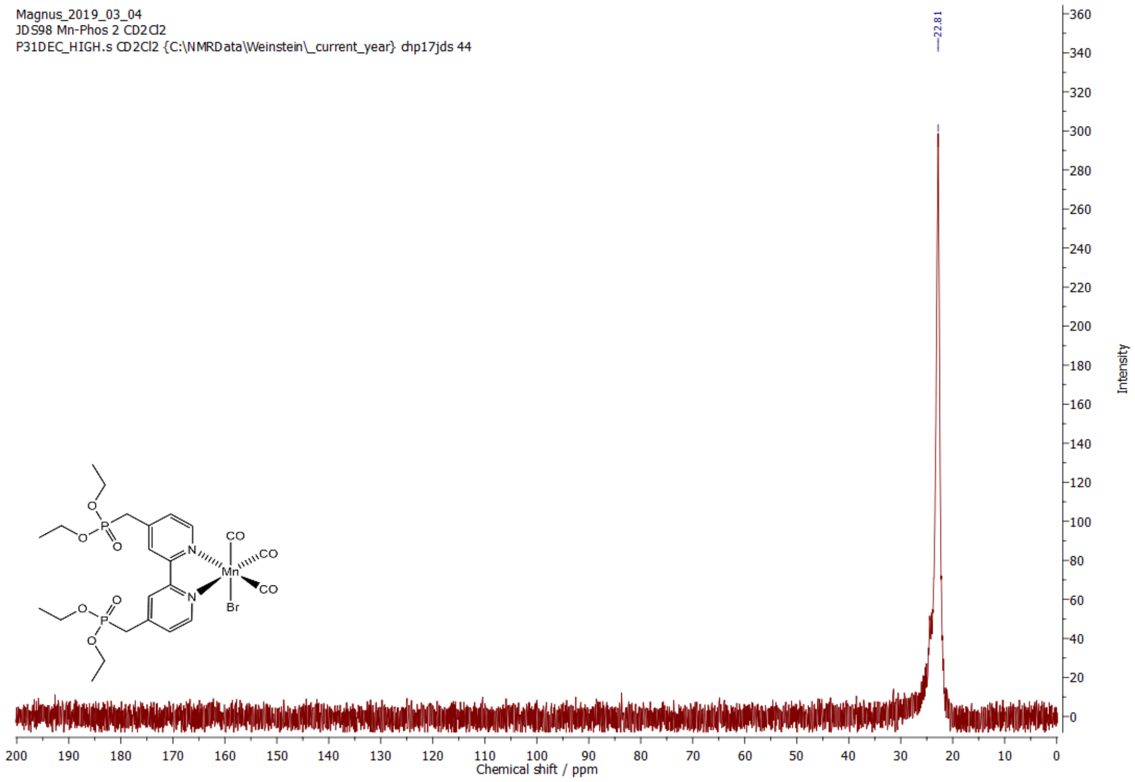

Figure S7 – ^{31}P NMR spectrum of [Mn(phos-bpy)(CO)$_3$Br] in d^2-dichloromethane.

Mass spectrum

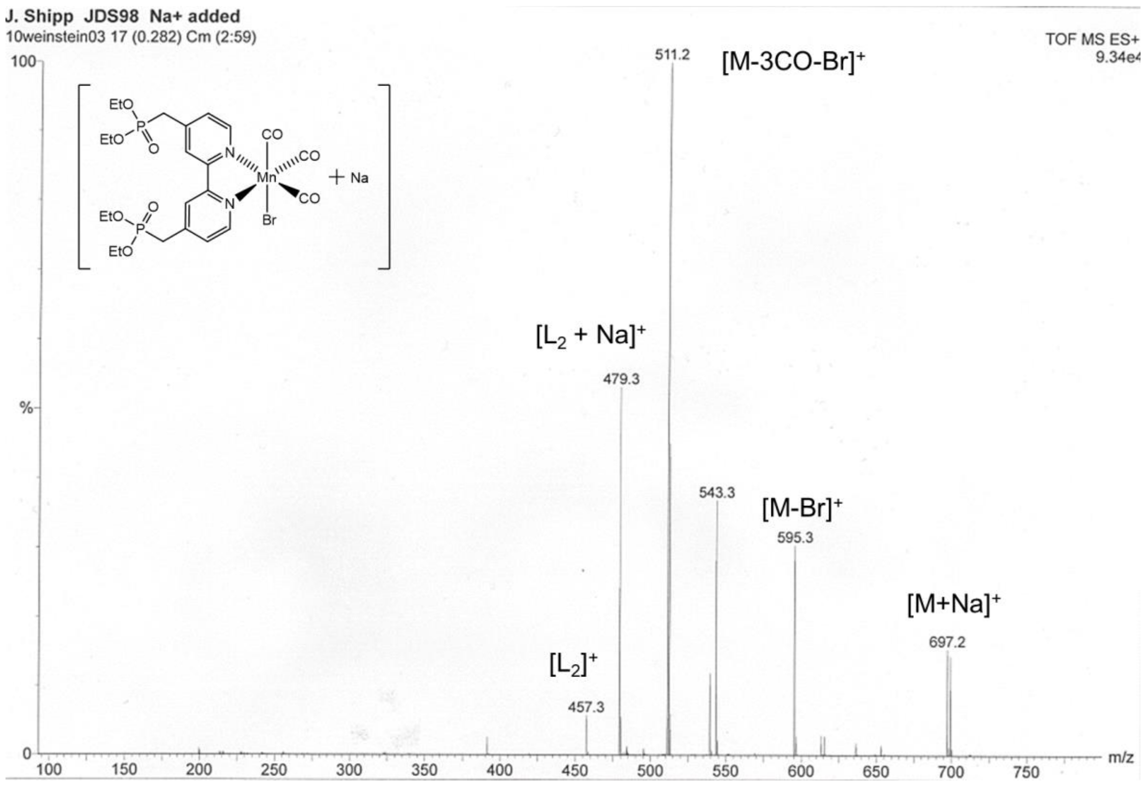

Figure S8 – Positive electrospray ionisation mass spectrum of [Mn(phos-bpy)(CO)$_3$Br], loaded by direct insertion with addition of sodium

Part 3: Additional electrochemical data

[Mn(phos-bpy)(CO)$_3$Br] – 1st oxidation

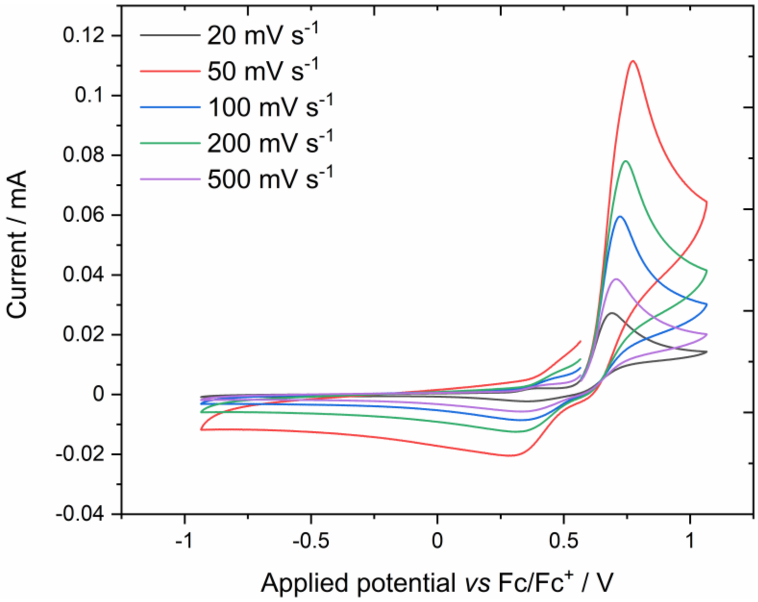

Figure S9 – Cyclic voltammograms of the 1st oxidation of [Mn(phos-bpy)(CO)$_3$Br] in anhydrous acetonitrile, under N_2 atmosphere at various scan rates, as shown on the graph.

[Mn(phos-bpy)$(CO)_3$Br] – 1st reduction

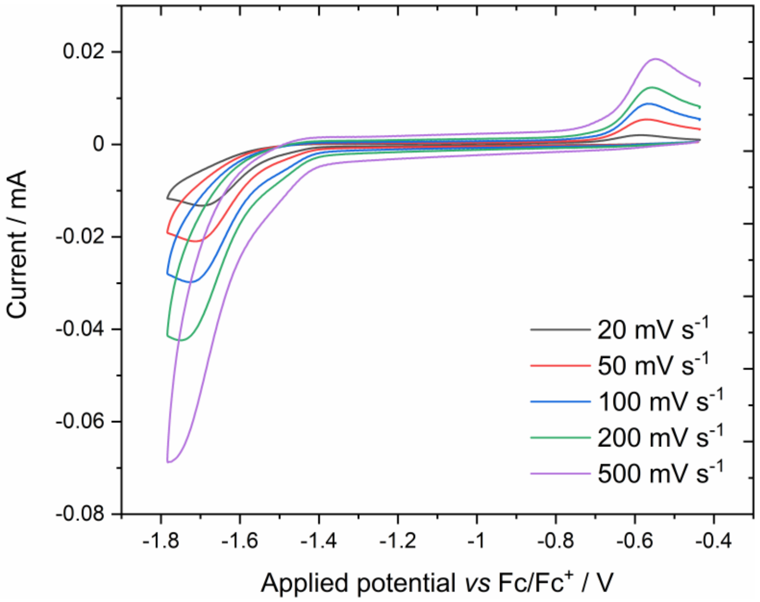

Figure S10 – *Cyclic voltammograms of the 1st reduction of [Mn(phos-bpy)$(CO)_3$Br] in anhydrous acetonitrile, under N_2 atmosphere at various scan rates, as shown on the graph.*

[Mn(phos-bpy)$(CO)_3$Br] – 2nd reduction

Due to the large $E_{p,a} – E_{p,c}$ separation of the second reduction process, it was not possible to isolate the redox process from the 1st reduction potential, thus the scan rate dependence of the second reduction was obtained from CV data in the range 0 to –2.5 V *vs.* Fc/Fc^+.

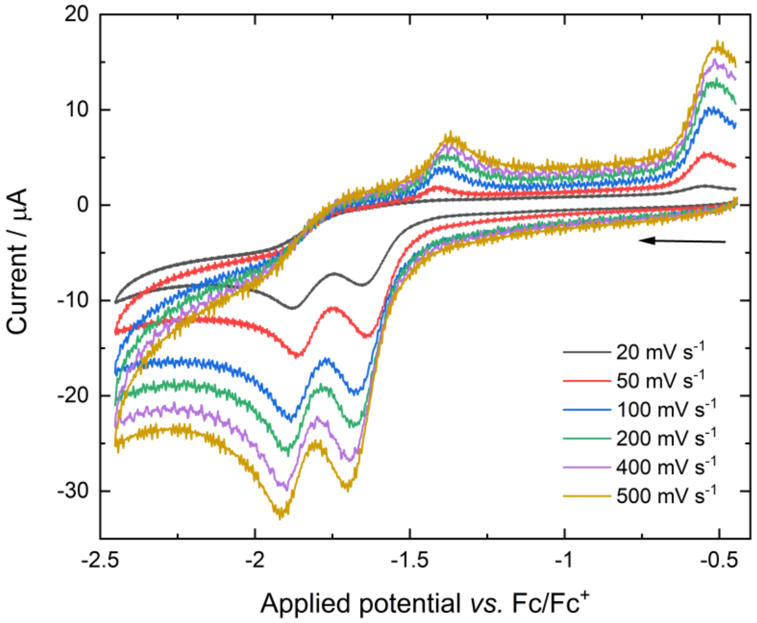

Figure S11 – Cyclic voltammograms of the 2nd reduction of [Mn(phos-bpy)$(CO)_3$Br] in anhydrous acetonitrile, under N_2 atmosphere at various scan rates, as shown on the graph.

[Mn(phos-bpy)(CO)$_3$Br] – Plots of peak current against square root of the scan rate

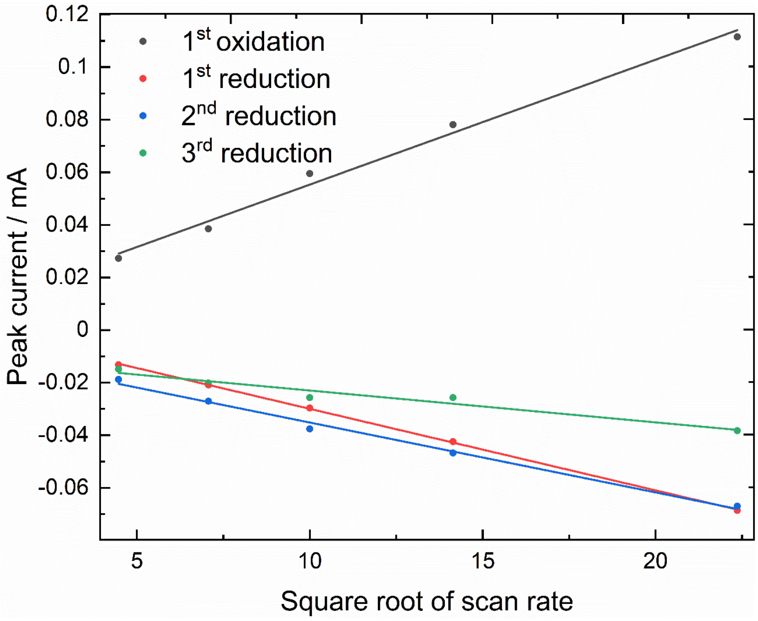

Figure S12 – Relationship between the total peak current of the redox couple against the square root of the scan rate for the four electrochemical processes of [Mn(phos-bpy)(CO)$_3$Br], recorded in anhydrous acetonitrile under N_2 atmosphere. The lines are linear fits of the data.

Part 4: Additional photocatalytic data

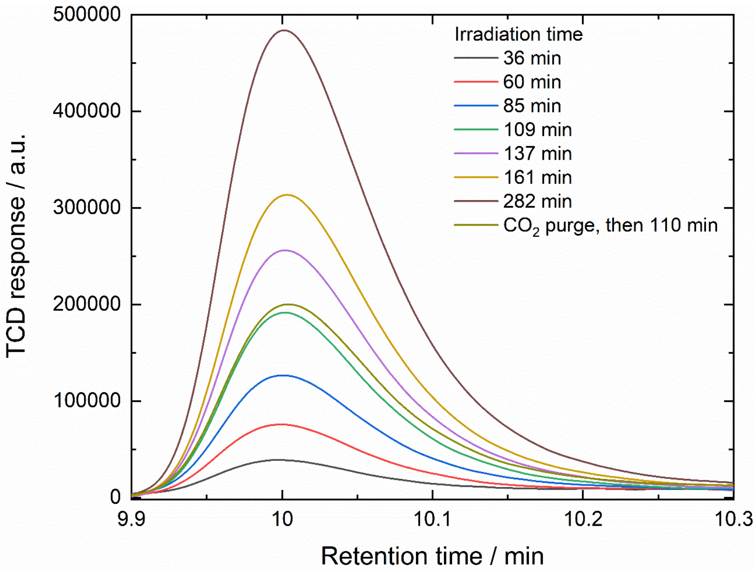

*Figure S133 – Gas chromatograms of the reaction mixture headspace during photocatalytic CO_2 reduction with **1**, **2**, and ascorbic acid in aqueous solution under 625 nm (308 mW cm^{-2}) irradiation.*

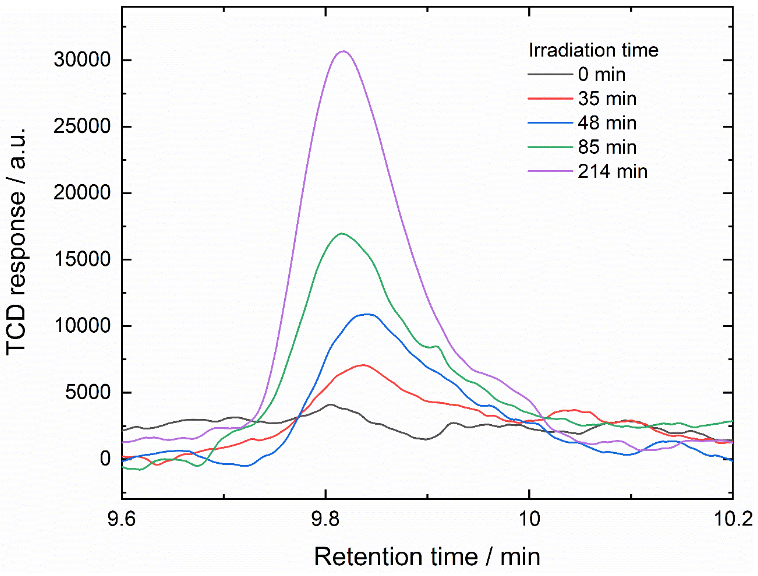

Figure S144 – Gas chromatography traces for the photocatalytic control experiment, where [Mn(phos-bpy)$(CO)_3$Br] and ascorbic acid were dissolved in aqueous solution and irradiated with 625 nm (308 mW cm^{-2}) light.

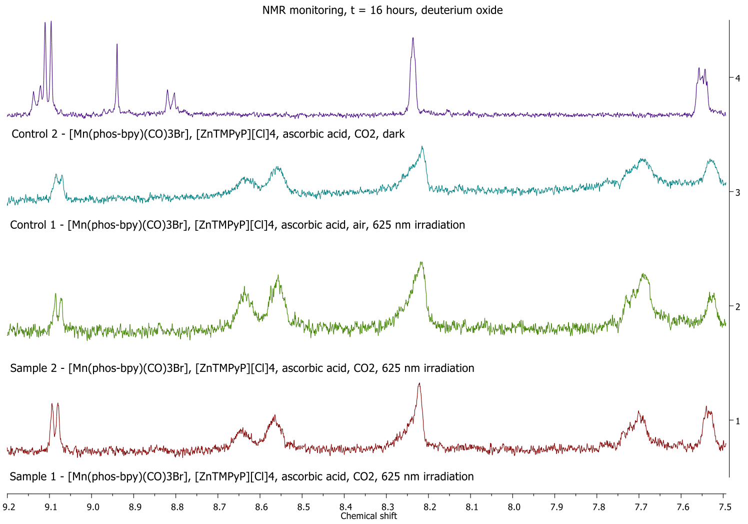

Figure S155 – ^{1}H NMR spectra of [Mn(phos-bpy)$(CO)_3$Br], [ZnTMPyP][Cl]$_4$, and ascorbic acid in D_2O under various conditions, recorded 16 hours after sample preparation. The irradiation intensity of the 625 nm light was 308 mW cm^{-2}.

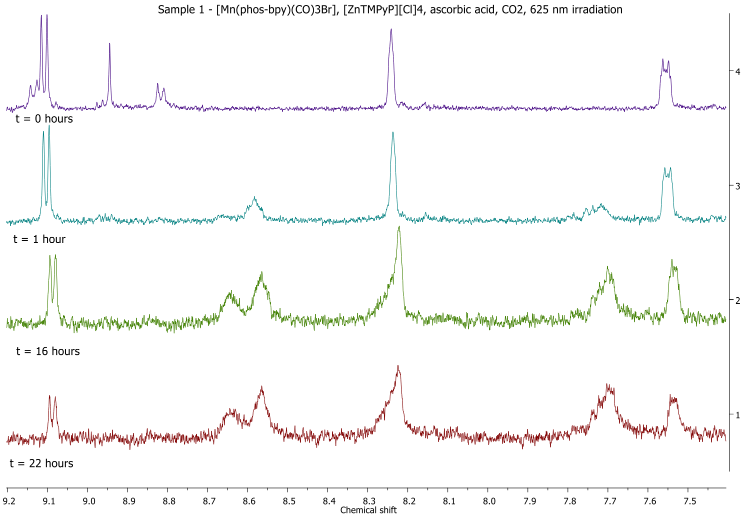

Figure S16 – ^{1}H NMR spectra of [Mn(phos-bpy)$(CO)_3$Br], [ZnTMPyP][Cl]$_4$, and ascorbic acid in D_2O under $^{12}CO_2$ atmosphere after at different irradiation times under 625 nm (308 mW cm^{-2}) light, as stated on the graph.

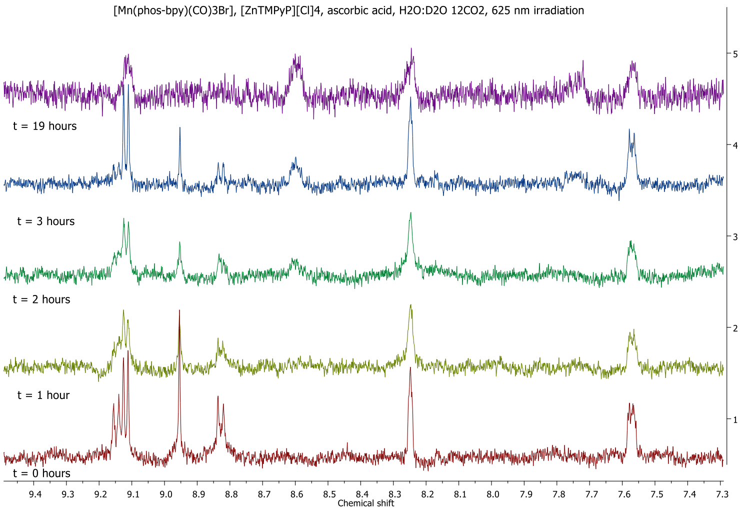

Figure S17 – ^{1}H NMR spectra of [Mn(phos-bpy)$(CO)_3$Br], [ZnTMPyP][Cl]$_4$, and ascorbic acid in 90:10 H_2O:D_2O under $^{12}CO_2$ atmosphere after at different irradiation times under 625 nm (308 mW cm^{-2}) light, as stated on the graph.

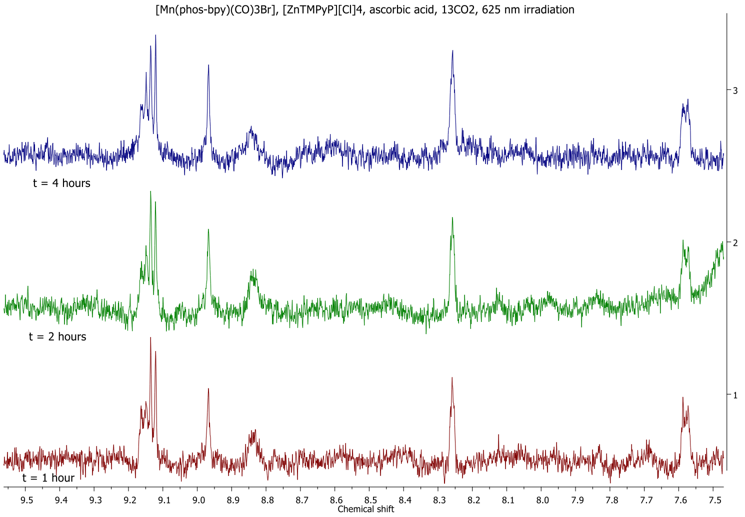

Figure S18 – ^{1}H NMR spectra of [Mn(phos-bpy)$(CO)_3$Br], [ZnTMPyP][Cl]$_4$, and ascorbic acid in 90:10 H_2O:D_2O under $^{13}CO_2$ atmosphere after at different irradiation times under 625 nm (308 mW cm^{-2}) light, as stated on the graph.

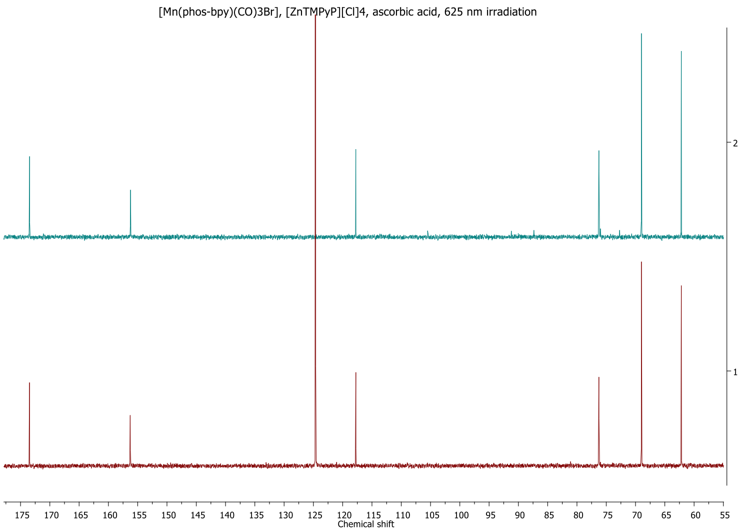

Figure S19 – $^{13}C\{^{1}H\}$ *NMR spectra of [Mn(phos-bpy)(CO)$_3$Br], [ZnTMPyP][Cl]$_4$, and ascorbic acid in 90:10 H_2O:D_2O under 3 hours of 625 nm irradiation (308 mW cm^{-2}). Top: Sample prepared without CO_2 purge. Bottom: Sample purged with $^{13}CO_2$. The $^{13}CO_2$ resonance was observed at 124 ppm.*

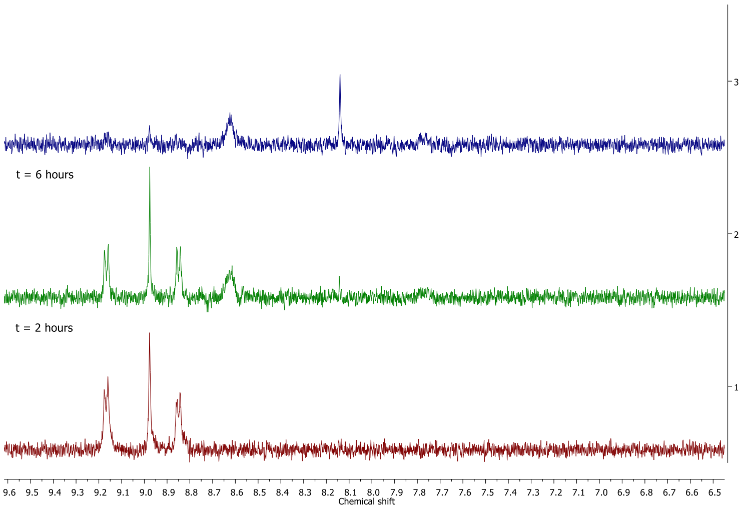

Figure S19 – ^{1}H *NMR spectra of [ZnTMPyP][Cl]$_4$ and ascorbic acid in D_2O at different irradiation times with 625 nm (308 mW cm^{-2}) light, as stated on the graphs*

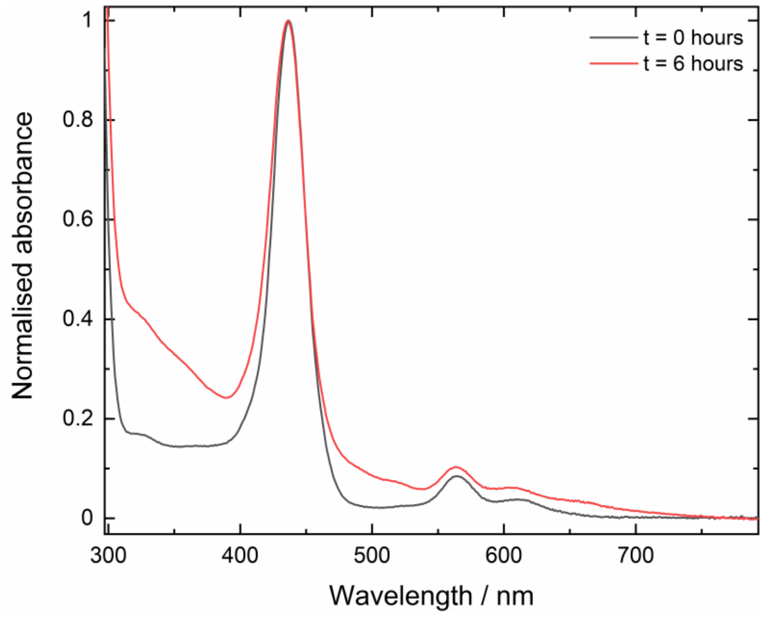

Figure S20 – UV-vis absorption spectra of [ZnTMPyP][Cl]$_4$ and ascorbic acid in D_2O at different irradiation times with 625 nm (308 mW cm^{-2}) light, as stated on the graph.

X-Ray crystallographic data

1 was crystallised by diffusion of Et_2O vapour into a solution of the complex in dichloromethane (DCM) to yield yellow crystals of the complex. The studied crystal with dimensions 0.5 x 0.5 x 0.4 mm was found to be triclinic with the P-1 space group. The unit cell contained two molecules of **1**, and no solvent co-crystallised with the complex. The complex formed the expected facial isomer, consistent with previously reported $[Mn(L_2)(CO)_3(X)]$ complexes. In the unit cell, the two molecules are offset and rotated 180° from one another, with the axial plane of the Mn centre pointed toward the bipyridyl π-system of the other complex. This geometry minimised the steric interaction of the four phosphonate ester groups by separating them by the largest possible distance. The space group of the crystal, P-1 (No. 2) was the same as the previously reported X-ray structure for $[Mn(4,4'\text{-}\{Et_2O_3PCH_2\}_2\text{-bpy})(CO)_3Br]$.[4] However, differences in the orientation of the two complexes in the unit cell structure were found, where the unit cell volume of the crystal data reported in this work was smaller than the previously reported crystal by 34.1 Å^3.

Crystal structure refinement procedure

Data were corrected for absorption using empirical methods (SADABS)[5] based upon symmetry equivalent reflections combined with measurements at different azimuthal angles.[6] The crystal structures were solved and refined against F2 values using ShelXT[7] for solution and ShelXL[8] for refinement accessed via the Olex2 program.[9] Non-hydrogen atoms were refined anisotropically. Hydrogen atoms were placed in calculated positions with idealized geometries and then refined by employing a riding model and isotropic displacement parameters.

The crystallographic information file is available in the CCSD, deposition number: 2119883

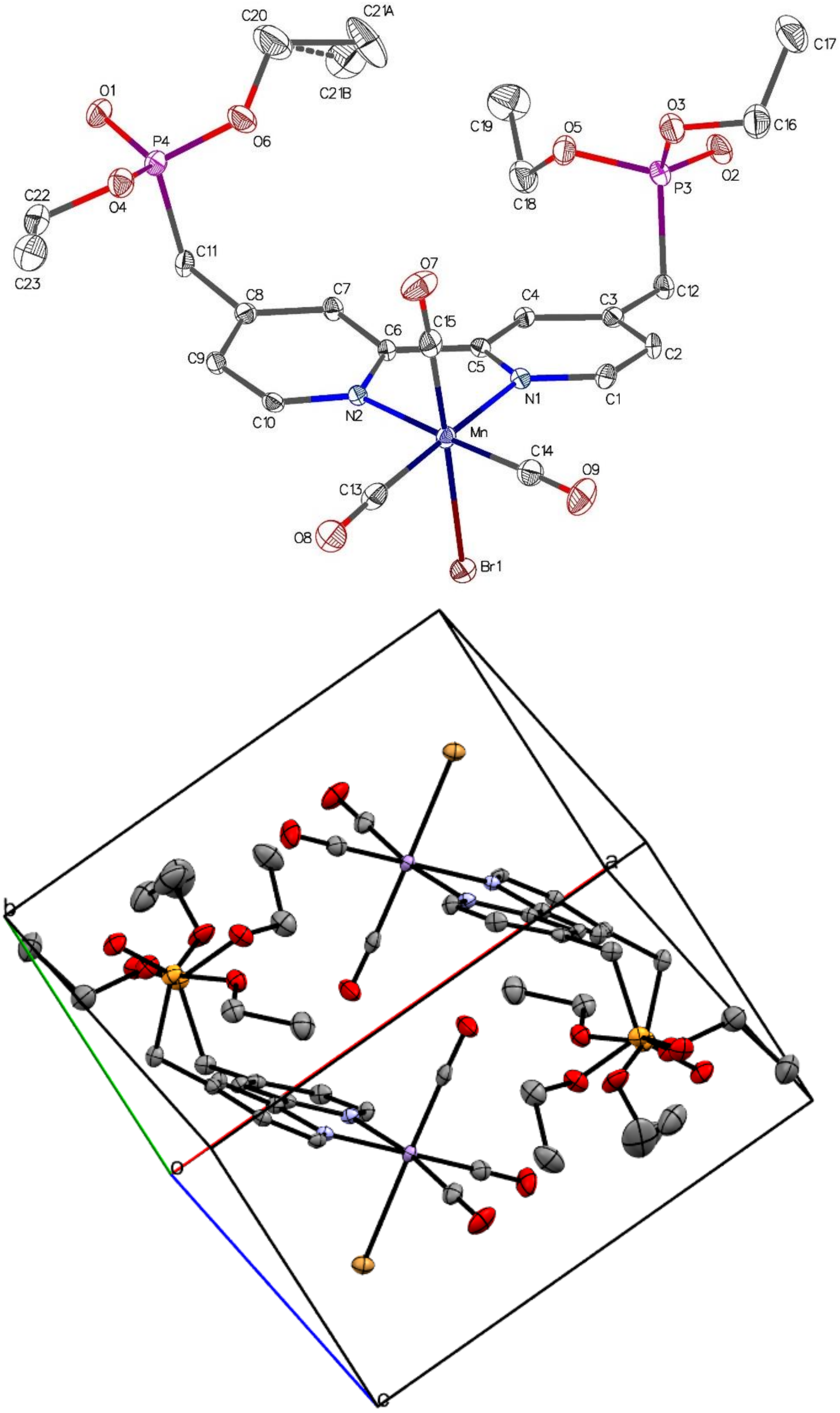

Figure S21 – Molecular structure and unit cell of the [Mn(4,4′-{$Et_2PO_3CH_2$}-2,2′-bipyridyl)$(CO)_3$Br] crystal, measured by single crystal X-ray diffraction, thermal ellipsoids are drawn at the 50% probability level.

Estimation of the apparent quantum yield of photocatalysis

The quantum yield of photocatalysis was estimated as the ratio of the number of moles of photons emitted from the LED diode to the number of moles of carbon monoxide formed from the catalytic reaction. The photon energy of the 625 nm light was 3.18×10^{-19} J, thus if the power density is 308 mW cm^{-2} and the light is focussed to a 4 cm^2 area, the

photon number per second is 3.87×10^{18} s^{-1}. Conversion to moles of photons per hour yields 1.79×10^{-9} mol hr^{-1}. The estimated quantum yields are shown in the table below.

$$Estimated\ quantum\ yield = \frac{Moles\ of\ CO\ produced\ (mol\ hr^{-1})}{Moles\ of\ incident\ photons\ (mol\ hr^{-1})} \cdot 100\ \%$$

Equation S1 – Estimation of the apparent quantum yield of photocatalysis based on the number of moles of product formed per unit time divided by the number of photons incident on the reaction mixture.

Table S1 – Estimated values for the apparent quantum yield of photocatalysis

**Irradiation time / hr**	**Moles of CO formed / mol**	**Moles of CO per hour / hr^{-1}**	**Estimated quantum yield (%)**
0.41	2.79×10^{-8}	6.70×10^{-8}	2.67
0.817	8.91×10^{-8}	8.91×10^{-8}	2.01
1.23	1.29×10^{-7}	1.04×10^{-7}	1.71
1.63	2.01×10^{-7}	1.23×10^{-7}	1.45
2.10	2.81×10^{-7}	1.34×10^{-7}	1.34
2.50	3.45×10^{-7}	1.38×10^{-7}	1.29
4.02	5.43×10^{-7}	1.35×10^{-7}	1.32

Estimation of the Gibbs energy of electron transfer

The thermodynamic favourability of the photosensitisation of $[MnBr(phos\text{-}bpy)(CO)_3]$ by $[ZnTMPyP][Cl]_4$ in the presence of ascorbic acid sacrificial donor was estimated with the Weller equation.

$$\Delta G_{et} = E^{\frac{1}{2}}\left(\frac{D}{D^{+}}\right) - E^{\frac{1}{2}}\left(\frac{A}{A^{-}}\right) - E_{00} - \frac{e^2}{\varepsilon d}$$

Equation S1 – Rehm-Weller equation for estimation of the Gibbs energy of electron transfer (ΔG_{et}) [D – electron donor, A – electron acceptor, $E^{1/2}$ – electrochemical half-wave potential, E_{00} – Excited/ground state energy difference, $\frac{e^2}{\varepsilon d}$ – coulombic term for electrostatic interaction].

The redox potentials of $[MnBr(phos\text{-}bpy)(CO)_3]$ were measured by cyclic voltammetry, and the E_{00} value for $[ZnTMPyP]Cl_4$ following 430 nm excitation was estimated by emission spectroscopy as 1.85 eV. Literature values were used for the redox potential of ascorbic acid (+1.09 V vs. Fc/Fc^{+}) oxidation, $[ZnTMPyP]Cl_4$ oxidation (+1.81 V vs. Fc/Fc^{+}), and $[ZnTMPyP]Cl_4$ reduction (-0.85 V vs. Fc/Fc^{+}).[10–12]

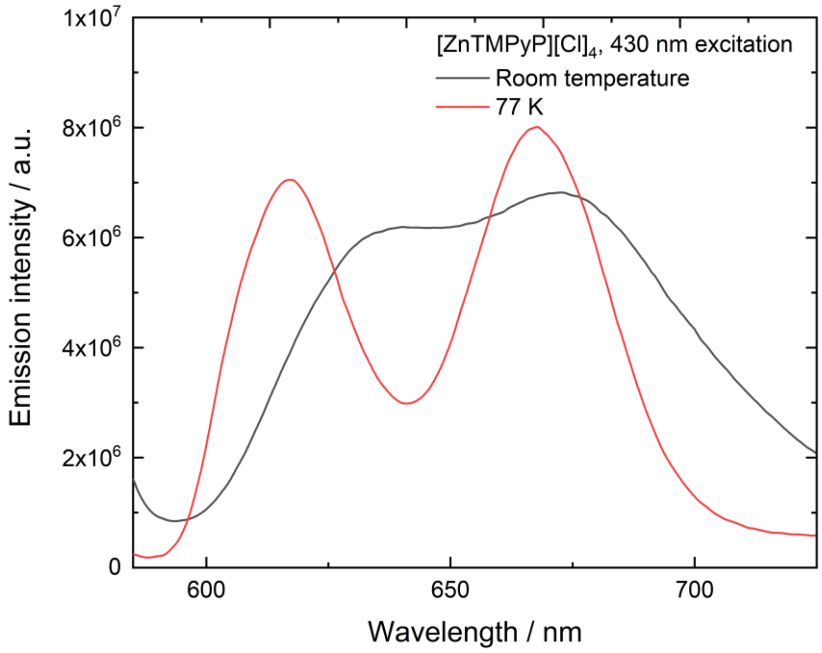

Figure S22 – Emission spectra of a solution of [ZnTMPyP][Cl]$_4$ in ethanol at room temperature and at 77 K,, used for estimation of the E_{00} transition energy.

Reductive quenching

It was found that the reductive quenching mechanism was not feasible, as the initial reduction of [ZnTMPyP]Cl_4 by ascorbic acid was thermodynamically unfavourable.

i) $ZnTMPyP + hv \rightarrow [ZnTMPyP]^{*}$

ii) $[ZnTMPyP]^{*} + Ascorbic\ acid \rightarrow [ZnTMPyP]^{-} + Ascorbic\ acid^{+}$

ii) $\Delta G_{et}\ =\ 1.09\ V - -0.85\ V -\ 1.85\ eV\ = 0.1\ V$

Equation S2 – Estimation of the Gibbs energy of electron transfer for the reduction of the [ZnTMPyP][Cl]$_4$ triplet state by ascorbic acid.

Oxidative quenching

The oxidative quenching of [MnBr(phos-bpy)(CO)$_3$] to form the active catalyst, [Mn(phos-bpy)(CO)$_3$]$^{-}$ was estimated to be thermodynamically unfavourable.

i) $ZnTMPyP + hv \rightarrow [ZnTMPyP]^{*}$

ii)$[ZnTMPyP]^{*} + [MnBr(phosbpy)(CO)_3] \rightarrow [ZnTPP]^{+} + [Mn(phosbpy)(CO)_3]^{-}$

iii) $[ZnTMPyP]^{*} + [Mn(phosbpy)(CO)_3] \rightarrow [ZnTMPyP]^{+} + [MnBr(phosbpy)(CO)_3]^{-}$

ii) $\Delta G_{et}\ =\ 1.81\ V -\ -1.73\ V -\ \ 1.85\ eV\ =\ 1.69\ V$

iii) $\Delta G_{et}\ =\ 1.81\ V -\ -1.86\ V -\ 1.85\ eV\ =\ 1.82\ V$

Equation S3 – Estimation of ΔG_{et} for the photoinduced electron transfer processes required for formation of [Mn(phos-bpy)(CO)$_3$]$^{-}$ by photosensitisation.

References:

(1) Miyatani, R.; Amao, Y. Visible Light-Induced Formic Acid Synthesis from HCO3−with Formate Dehydrogenase and Water-Soluble Zinc Porphyrin. *J. Japan Pet. Inst.* **2009**, *52* (6), 322–331. https://doi.org/10.1627/jpi.52.322.

(2) Steinlechner, C.; Roesel, A. F.; Oberem, E.; Päpcke, A.; Rockstroh, N.; Gloaguen, F.; Lochbrunner, S.; Ludwig, R.; Spannenberg, A.; Junge, H.; Francke, R.; Beller, M.; Steinlechner, C.; Roesel, A. F.; Oberem, E.; Pa, A.; Lochbrunner, S.; Ludwig, R.; Spannenberg, A.; Junge, H.; Francke, R.; Beller, M. Selective Earth-Abundant System for CO 2 Reduction: Comparing Photo- and Electrocatalytic Processes. *ACS Catal.* **2019**, *9* (3), 2091–2100. https://doi.org/10.1021/acscatal.8b03548.

(3) Penicaud, V.; Odobel, F.; Bujoli, B. Facile and Efficient Syntheses of 2,2'-Bipyridine-Based Bis(Phosphonic) Acids. *Tetrahedron Lett.* **1998**, *39*, 3689–3692.

(4) Woo, S. J.; Choi, S.; Kim, S. Y.; Kim, P. S.; Jo, J. H.; Kim, C. H.; Son, H. J.; Pac, C.; Kang, S. O. Highly Selective and Durable Photochemical CO 2 Reduction by Molecular Mn(I) Catalyst Fixed on a Particular Dye-Sensitized TiO 2 Platform. *ACS Catal.* **2019**, *9* (3), 2580–2593. https://doi.org/10.1021/acscatal.8b03816.

(5) Bruker. SADABS. Bruker Axs Inc.: Madison, Wisconsin, USA 2016.

(6) Krause, L.; Herbst-Irmer, R.; Sheldrick, G. M.; Stalke, D. Comparison of Silver and Molybdenum Microfocus X-Ray Sources for Single-Crystal Structure Determination. *J. Appl. Crystallogr.* **2015**, *48* (1), 3–10. https://doi.org/10.1107/S1600576714022985.

(7) Sheldrick, G. M. SHELXT - Integrated Space-Group and Crystal-Structure Determination. *Acta Crystallogr. Sect. A Found. Crystallogr.* **2015**, *71* (1), 3–8. https://doi.org/10.1107/S2053273314026370.

(8) Sheldrick, G. M. Crystal Structure Refinement with SHELXL. *Acta Crystallogr. Sect. C Struct. Chem.* **2015**, *71* (Md), 3–8. https://doi.org/10.1107/S2053229614024218.

(9) Dolomanov, O. V.; Bourhis, L. J.; Gildea, R. J.; Howard, J. A. K. K.; Puschmann, H. OLEX2: A Complete Structure Solution, Refinement and Analysis Program. *J. Appl. Crystallogr.* **2009**, *42* (2), 339–341. https://doi.org/10.1107/S0021889808042726.

(10) Pellegrin, Y.; Odobel, F. Les Donneurs d'électron Sacrificiels Pour La Production de Combustible Solaire. *Comptes Rendus Chim.* **2017**, *20* (3), 283–295. https://doi.org/10.1016/j.crci.2015.11.026.

(11) Harriman, A.; Porter, G.; Walters, P. Photo-Oxidation of Metalloporphyrins in Aqueous Solution. *J. Chem. Soc. Faraday Trans. 1* **1983**, *79*, 1335–1350.

(12) Ponce, C. P.; Steer, R. P.; Paige, M. F. Photophysics and Halide Quenching of a Cationic Metalloporphyrin in Water. *Photochem. Photobiol. Sci.* **2013**, No. 12, 1079–1085. https://doi.org/10.1039/c3pp50022e.
